# MyD88 in myofibroblasts enhances nonalcoholic fatty liver disease-related hepatocarcinogenesis via promoting macrophage M2 polarization

**DOI:** 10.1186/s12964-024-01489-x

**Published:** 2024-01-30

**Authors:** Yu Liu, Haiqiang Chen, Xuanxuan Yan, Jie Zhang, Zhenzhong Deng, Maosheng Huang, Jianchun Gu, Jinhua Zhang

**Affiliations:** 1 College of Life Science and Bioengineering, Beijing Jiaotong University, No.3 Shangyuancun Road, Beijing, 100044 P.R. China; 2 Department of Oncology, Xinhua Hospital, Shanghai Jiaotong University School of Medicine, 1665 Kongjiang Road, Shanghai, 200092 P. R. China; 3 Department of Epidemiology, The University of Texas MD Anderson Cancer Center, Houston, TX USA

**Keywords:** MyD88, Nonalcoholic fatty liver disease, Hepatocellular carcinoma, Macrophage polarization, CCL9/CCL15

## Abstract

**Background:**

Nonalcoholic fatty liver disease (NAFLD) is a major cause of chronic liver diseases and has emerged as the leading factor in the pathogenesis of hepatocellular carcinoma (HCC). MyD88 contributes to the development of HCC. However, the underlying mechanism by which MyD88 in myofibroblasts regulates NAFLD-associated liver cancer development remains unknown.

**Results:**

Myofibroblast MyD88-deficient (SMA^MyD88−/−^) mice were protected from diet-induced obesity and developed fewer and smaller liver tumors. MyD88 deficiency in myofibroblasts attenuated macrophage M2 polarization and fat accumulation in HCC tissues. Mechanistically, MyD88 signaling in myofibroblasts enhanced CCL9 secretion, thereby promoting macrophage M2 polarization. This process may depend on the CCR1 receptor and STAT6/ PPARβ pathway. Furthermore, liver tumor growth was attenuated in mice treated with a CCR1 inhibitor. CCLl5 (homologous protein CCL9 in humans) expression was increased in myofibroblasts of HCC and was associated with shorter survival of patients with HCC. Thus, our results indicate that MyD88 in myofibroblasts promotes NAFLD-related HCC progression and may be a promising therapeutic target for HCC treatment.

**Conclusion:**

This study demonstrates that MyD88 in myofibroblasts can promote nonalcoholic fatty liver disease-related hepatocarcinogenesis by enhancing macrophage M2 polarization, which might provide a potential molecular therapeutic target for HCC.

**Graphical Abstract:**

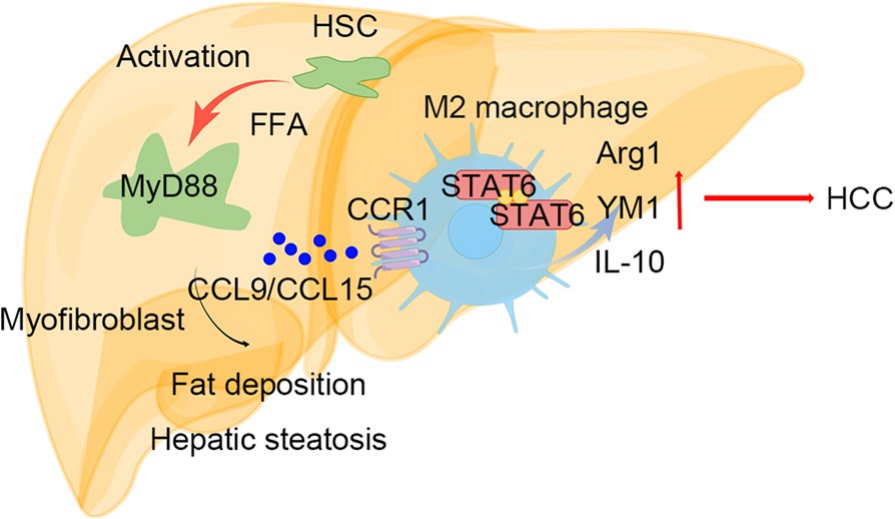

**Supplementary Information:**

The online version contains supplementary material available at 10.1186/s12964-024-01489-x.

## Introduction

Liver cancer is one of the most common cancers and the third leading cause of cancer-related deaths worldwide [1]. Hepatocellular carcinoma (HCC) is the most prevalent subtype of liver cancer, accounting for approximately 90% of primary liver cancers. HCC frequently occurs in patients with underlying chronic liver disease, mainly caused by hepatitis B and hepatitis C virus infections, excessive alcohol consumption, and nonalcoholic fatty liver disease (NAFLD) [2]. Recent epidemiological studies have shown that the annual incidence of NAFLD-associated HCC is increasing [3, 4]. NAFLD encompasses a spectrum of liver pathologies, ranging from simple steatosis (NAFL), nonalcoholic steatohepatitis (NASH) and fibrosis/cirrhosis; these conditions can progress to HCC [5]. Thus, it is crucial to obtain a better understanding of the obesity-associated tumor-promoting mechanisms underlying HCC development.

The tumor microenvironment (TME) includes various stromal cells, such as fibroblasts, endothelial cells, adipocytes and immune cells, as well as noncellular components, which are known to play critical roles in tumor progression and therapeutic responses [6, 7]. Among TME stromal cells, cancer-associated fibroblasts (CAFs) are one of the most abundant cellular components [8, 9]. Hepatic stellate cells (HSCs) are the main source of CAFs in HCC. Activated HSCs, highly express α-smooth actin (α-SMA), act as fibroblasts, produce ECM proteins, and play a key role in liver fibrosis [10]. CAFs contribute to HCC development by reducing immune surveillance, promoting tumor angiogenesis, and secreting regulatory molecules, and particularly, synthesizing and remodeling the ECM [11, 12]. The progression of NAFLD to HCC is characterized by inflammation and fibrosis. Hepatic injuries eventually lead to HSCs activation, collagen deposition, and ECM deposition, consequently promoting fibrosis and cirrhosis [13, 14]. However, the mechanism by which HSCs promote NAFLD and lead to HCC development remains poorly understood.

Toll-like receptors (TLRs) are pattern recognition receptors that mediate the recognition of pathogen-associated molecular patterns [15]. Myeloid differentiation factor 88 (MyD88), a ligand of TLRs, is a signaling adaptor protein shared by all TLRs, except for TLR3, that activates proinflammatory cascades [16, 17]. MyD88 deletion increases the risk of developing type 2 diabetes and hepatic steatosis in animals fed with a high-fat diet (HFD) [18, 19]. Furthermore, hepatocyte-specific deletion of MyD88 is a predisposing factor for glucose intolerance, inflammation, and hepatic insulin resistance (IR) [20]. In contrast, deleting MyD88 in intestinal epithelial cells or myeloid cells protects mice from obesity and IR [18, 21, 22]. MyD88 also promotes the development of diethylnitrosamine (DEN)-induced hepatocarcinogenesis [23]. However, the contributions of MyD88 to NAFLD-related HCC have not yet been reported. MyD88 is widely expressed in various liver cells, including hepatocytes, HSCs, and Kupffer cells. In myofibroblasts, MyD88 inhibits hepatocarcinogenesis by preventing aerobic glycolysis of liver cancer cells in DEN/CCl_4_-induced HCC [24]. However, there is limited information about the function of MyD88 in myofibroblasts in NAFLD and NAFLD-related liver cancer.

In the present study, we explored the HSC-specific role of TLR signaling in NAFLD-related HCC development using mice with a genetic deletion of MyD88 in myofibroblasts. Myofibroblast-specific MyD88 deletion decreased fat accumulation and tumor incidence in HFD-induced NAFLD and DEN/HFD-induced liver cancer.

## Materials and methods

### Mice

MyD88^fl/fl^ and α-SMA-cre mice on a C57BL/6 background have been described previously [24, 25]. Mice with a conditional knockout of MyD88 in α-SMA-expressing myofibroblasts (SMA^MyD88−/−^) were generated by crossing MyD88^fl/fl^ and α-SMA-cre mice. Control mice were cre-negative littermates. All mice were maintained in specific pathogen-free and humidity- and temperature-controlled microisolator cages with a 12-h light/dark cycle at the Institute of Biophysics, Chinese Academy of Sciences. All animal studies were performed after being approved by the Institutional Laboratory Animal Care and Use Committee of the Beijing Jiaotong University.

### HFD-induced nonalcoholic fatty liver

Male mice were fed normal diet (ND, 10% of total energy from fat, Huafukang, Beijing, CN) or were fed HFD (60% of total energy from fat, Huafukang, Beijing, CN) beginning at 6 weeks of age to induce obesity for 12 weeks.

### DEN/HFD-induced hepatocellular carcinoma

The male mice were i.p. injected with DEN (50 µg/g body weight) (Sigma-Aldrich, St. Louis, MO, USA) at the age of 15 days and were fed HFD (60% of total energy from fat, Huafukang, Beijing, CN) beginning at 6 weeks of age. Liver tumorigenesis was monitored for 10 months.

### Mouse transplanted tumor model

Groups of MyD88^fl/fl^ and SMA^MyD88−/−^ mice were fed with HFD for 3 months. Exponentially growing Hepa1-6 cells were harvested and washed, and a suspension of 1 × 10^6^ cells in 200 µl of sterilized phosphate-buffered saline was injected subcutaneously into the abdomen region of MyD88^fl/fl^ and SMA^MyD88−/−^ mice. The mice were continued fed with HFD. Two days later, the mice received vehicle or 3 mg/kg CCR1 inhibitor J113863 by intraperitoneal injection twice a week for 2 weeks. Tumor growths were measured for 2 weeks.

### Blood biochemical assays

Blood samples of mice were centrifuged at 3000 rpm for 8 min to obtain serum. The levels of serum alanine aminotransferase (ALT), aspartate aminotransferase (AST), triglyceride (TG) and total cholesterol (TC) were detected by Beijing Vital River Laboratory Animal Technology (Beijing, China).

For the glucose tolerance test (GTT), blood samples were obtained at 0, 15, 30, 60 and 120 min after intraperitoneal injection of 2 g/kg dextrose. Blood glucose values were determined using Accu-Chek Performa glucometer (Roche, Basel, Switzerland).

### Histology and immunostaining

Paraffin or cryostat sections of liver tissue were prepared as described previously [26]. The sliced liver paraffin sections were stained with hematoxylin and eosin (H&E) (Zhongshanjinqiao, Beijing, China). To detect hepatic fat accumulation, cryostat liver sections were stained with Oil Red O (Baso, Zhuhai, Guangzhou, CN). For immunohistochemistry (IHC), cryostat sections were incubated with anti-Ki67 antibodies (BD Pharmingen, San Diego, CA, USA) followed by incubation with horseradish peroxidase (HRP)-conjugated secondary antibodies. For fluorescence staining, cryostat sections were incubated with anti-F4/80, anti-CD11b, anti-Gr1, anti-CD86 (Santa Cruz Biotechnology, TX, USA), anti-CD206 antibodies (BD Pharmingen, San Diego, CA, USA), anti-MyD88 and anti-α-SMA antibodies (Abcam, Cambridge, Cambs, UK) followed by incubation with Alexa Fluor 488-conjugated or Alexa Fluor 594-conjugated secondary antibodies (1:500; Invitrogen, Carlsbad, CA, USA). Sections were evaluated under a micro-scope (DP71, OLYMPUS, Tokyo, Japan) for bright-field and fluorescence microscopy.

### Cell lines and treatments

The LX-2 cell line was purchased from Xiangya Medical College (Changsha, China), and RAW264.7 cell line was obtained from the American Type Culture Collection (ATCC; TIB-71, VA, USA). These cells were cultured in Dulbecco’s modified Eagle’s medium (DMEM)/1640 containing 10% fetal bovine serum (FBS) and 1% penicillin/streptomycin at 37 °C with 5% CO_2_. The LX-2 cells were exposed to ST2825 (10 µM, MedChemExpress, Princeton, NJ, USA) for 2 h. After incubation, the cells were challenged with OA (200 µM, Sigma-Aldrich, St. Louis, MO, USA) for 24 h for further analysis. RAW264.7 cells were induced by 20 ng/mL IL-4, 20 ng/mL IL-13, 200 µM OA, 20 ng/mL CCL9 recombinant protein, and 500 nM J113863 (inhibitor of CCL9 receptor CCR1) for 48 h.

#### Isolation of primary HSCs

Primary HSCs was isolated from livers as previously described using eight-week-old mice. The mice were anesthetized with 3% ethyl carbamate and treated with phosphate balanced solution and 0.16 mg/mL collagenase I for liver perfusion. The liver was removed, divided it into small pieces, and was placed in the digestive buffer at 37 °C for 30 min. The filtered cells were centrifuged at 50×g for 2 min to remove hepatocytes. For HSC enrichment, the remaining NPC fraction was resuspended in 11.5% OptiPrep (Axis-Shield, Oslo, NOR) and placed between a bottom cushion of 15% OptiPrep and a top layer of PBS [27]. After centrifugation at 1400×g for 20 min, the HSC fraction at the interface between the top and intermediate layers was obtained. The purity of the HSC fraction was estimated based on the autofluorescence signal.

### Cellular immunofluorescence

LX-2 cells were plated into 24-well plates. For fluorescence staining, the cells were fixed in 4% formaldehyde for 15 min and permeabilized with 0.2% Triton-X 100 for 10 min at room temperature. The cells were incubated with 2% BSA to block nonspecific binding sites. Then, the cells were incubated with anti-MyD88, anti-α-SMA antibodies (Abcam, Cambridge, Cambs, UK) and anti-CD206 antibodies (BD Pharmingen, San Diego, CA, USA), followed by incubation with Alexa Fluor 488-conjugated or Alexa Fluor 594-conjugated secondary antibodies (Invitrogen, Carlsbad, CA, USA).

### RNA sequencing analysis

RNA-sequencing analyses were performed in DEN/HFD-induced HCC tissues from control and SMA^MyD88−/−^ mice. Total RNA was extracted with RNeasy Mini Kit (QIAGEN, Dusseldorf, Germany), and RNA-sequencing analyses were performed on the BGISEQ-500 sequencer platform by BGI (Shenzhen, China). Stats package and plots with ggplot2 (RRID:SCR_014601) package in R (version 3.5) were used in principle component analysis. The raw transcriptomic reads were mapped to C57BL/6 genome using HISAT40/Bowtie241 tools after removing adaptor sequences, reads containing polyN sequences, and low-quality reads. Normalization was performed and RESM software was used. Significantly differentially expressed genes (DEGs) were identified by setting padj < 0.05, and the absolute value of log2 Ratio ≤ 0.5. The KEGG (Kyoto Encyclopedia of Genes and Genomes) enrichment analysis was performed by using phyper in R. All data mining, and figure presentation were conducted on the Dr Tom network platform of BGI (http://report.bgi.com).

### Western blot analysis

Western blot was performed as previously described. Tissue and cell extracts were separated by electrophoresis on a 10% SDS-PAGE gel at 65 V for 45 min and 115 V for 1 h 20 min, then transferred to a PVDF membrane at 200 mA for 1 h. Membranes were blocked with 5% milk in TBST for 2 h and incubated overnight at 4 °C with the following primary antibodies: anti-MyD88, anti-α-SMA (Abcam, Cambridge, Cambs, UK), anti-FASN (Santa Cruz Biotechnology, TX, USA), anti-SREBP1 (Santa Cruz Biotechnology, TX, USA), anti-SCD1 (Santa Cruz Biotechnology, TX, USA), anti-STAT6 and anti-p-STAT6 antibodies (Affinity Biosciences, Liyang, Jiangsu, CN). HRP-conjugated goat anti-mouse IgG and goat anti-rabbit IgG were used as secondary antibodies. Blots were scanned using a Clinx Science Instrument. All specific bands were quantified with the ImageJ (version 1.8 RRID:SCR_003070) automated Digitizing System.

### Quantitative real-time polymerase chain reaction (qPCR)

Total RNA was isolated respectively from frozen liver tissues and cells by TRIzol reagent (Invitrogen, USA), and then up to 1 µg of RNA was reverse transcribed by Prime Script RT Master Mix Kit (Tiangen Biotech, Beijing, CN). cDNA was duplicated by SYBR Premix ExTaqTM Kit (Tiangen Biotech, Beijing, CN). The primer sequences are listed in Supplementary Table 1. Data were analyzed using the 2^−ΔΔCt^ method and normalized to β-actin expression as previously described.

### Clinical samples

Primary human HCC and adjacent nontumor liver tissue samples were obtained from HCC patients at Xinhua Hospital Affiliated to Shanghai Jiaotong University School of Medicine, with informed consent obtained from all patients. The Ethics Committee of Beijing Jiaotong University approved the use of human specimens in accordance with the Declaration of Helsinki.

### Public database analysis

Gene expression data (GSE164760, GSE190967 profiling data) were downloaded as raw signals from the Gene Expression Omnibus (GEO, http://www.ncbi.nlm.nih.gov/geo), analyzed using the Geo2R function from NCBI (https://www.ncbi.nlm.nih.gov/geo/geo2r). The rank sum test was applied to evaluate the differences in the median CCL15 expression levels between patients with NASH or HCC and healthy controls. Pearson’s correlation analysis was performed on α-SMA and CCL15 or α-SMA and CCR1. Gene expression of HCC was obtained from published gene expression profiles included in the TCGA dataset (https://www.cancer.gov/). Survival analysis was performed with MyD88, α-SMA and CCL15, combined α-SMA/MyD88/CCR1 gene expression bifurcated by the spline method to group into low vs. high. Overall survival was calculated by the Kaplan-Meier method. Differences were analyzed by the log-rank test.

### Quantitative and statistical analysis

The data were expressed as means ± SEM and analyzed using GraphPad Prism (version 7.0; RRID:SCR_002798). Differences between the two groups were compared using two-tailed unpaired Student’s t-test analysis. Two-way ANOVA was used for multiple comparisons. For all tests, *p* < 0.05 was considered statistically significant. *p* < 0.01 was considered extremely significant.

## Results

### MyD88 expression in myofibroblasts was upregulated during the progression of NAFLD-related HCC

**Fig. 1:**
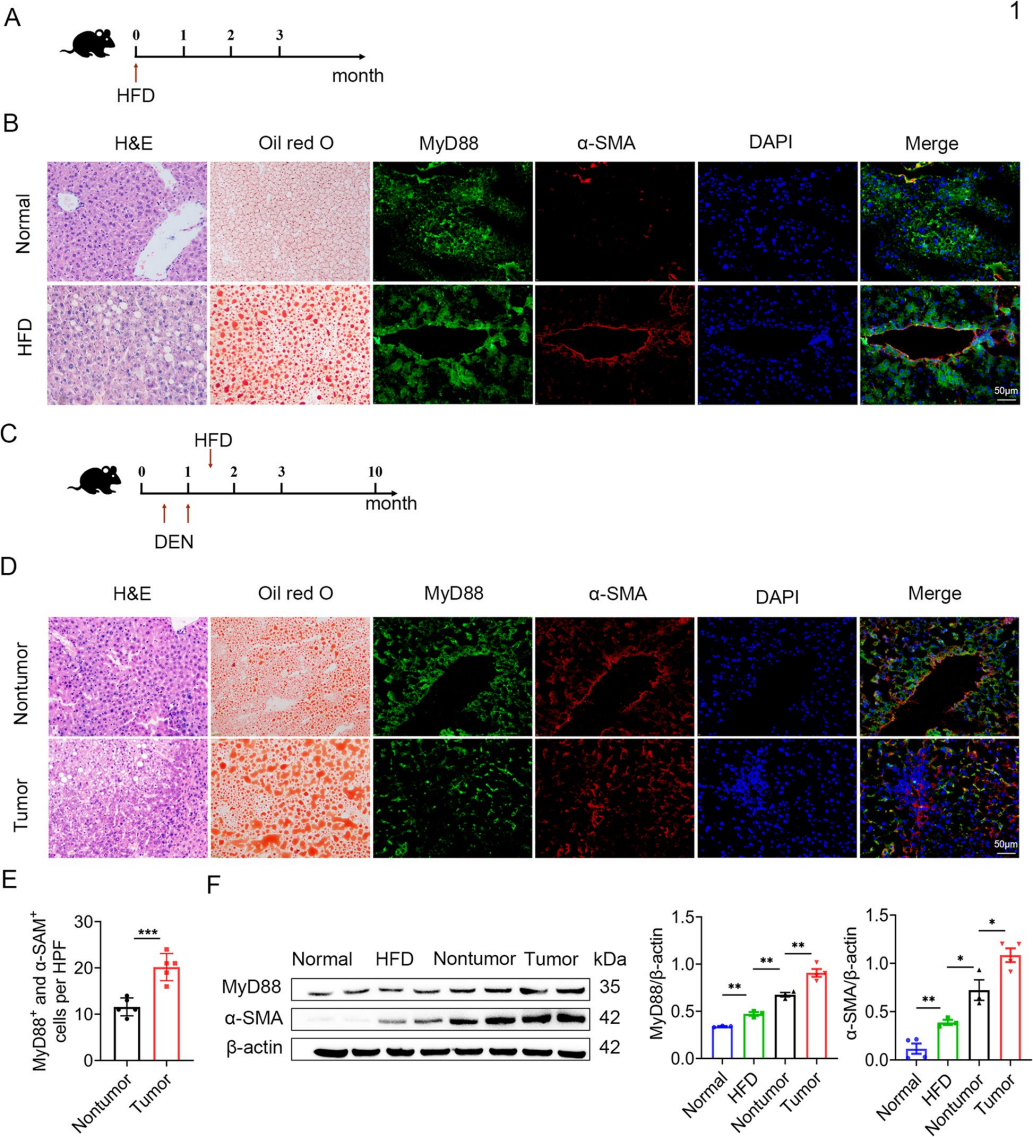
MyD88 expression in myofibroblasts was associated with NAFLD-related liver cancer.  C57BL/6 mice were used for NAFLD model ( *n*  = 5 per group) and liver cancer model ( *n*  = 10 per group). **A** Schematic representation of the HFD induced NAFLD model. **B** Representative staining of H&E, Oil red O, and double staining of α-SMA and MyD88 in normal liver and NAFLD samples. Scale bar, 50 μm. **C** Schematic representation of the DEN/HFD induced liver cancer model. **D** Representative staining of H&E, Oil red O, and double staining of α-SMA and MyD88 in normal and HCC samples. Scale bar, 50 μm. **E** Statistical analysis (magnification, 200×). *** *p*  < 0.001. **F** The expression levels of MyD88 in liver tissues were determined by western blot. The densities of proteins were quantified using densitometry. Proteins were normalized to β-actin. *p<0.05, **p<0.01.  HFD: High fat diet; NAFLD: Non-alcoholic fatty liver disease; DEN: N-nitrosodiethylamine

To explore the correlation between MyD88 expression in the mouse liver and development of NAFLD-related HCC, we used C57BL/6 mouse models of NAFLD and HFD-related HCC. We employed a short-term HFD model as an NAFLD model (Fig. 1A), and revealed an increasing aggravation of liver impairment in fatty liver tissues compared with normal liver tissues via H&E staining. Oil red O staining showed increased liver fat accumulation. Immunofluorescence staining indicated that MyD88 expression in α-SMA^+^ cells was significantly upregulated in fatty liver tissues (Fig. 1B). The DEN/HFD model is an NAFLD-related HCC model (Fig. 1C). Consistent with the fatty liver tissue results, fat accumulation, and the proportion of MyD88 and α-SMA double-positive cells were increased in HCC tissues (Fig. 1D-E). MyD88 and α-SMA protein levels (Fig. 1F) were consistent with immunofluorescence staining (Fig. 1D). Thus, MyD88 expression in myofibroblasts appears correlated with the pathogenesis of NAFLD-related HCC.

### MyD88 deficiency in myofibroblasts attenuated fat accumulation in HFD-induced NAFLD

**Fig. 2:**
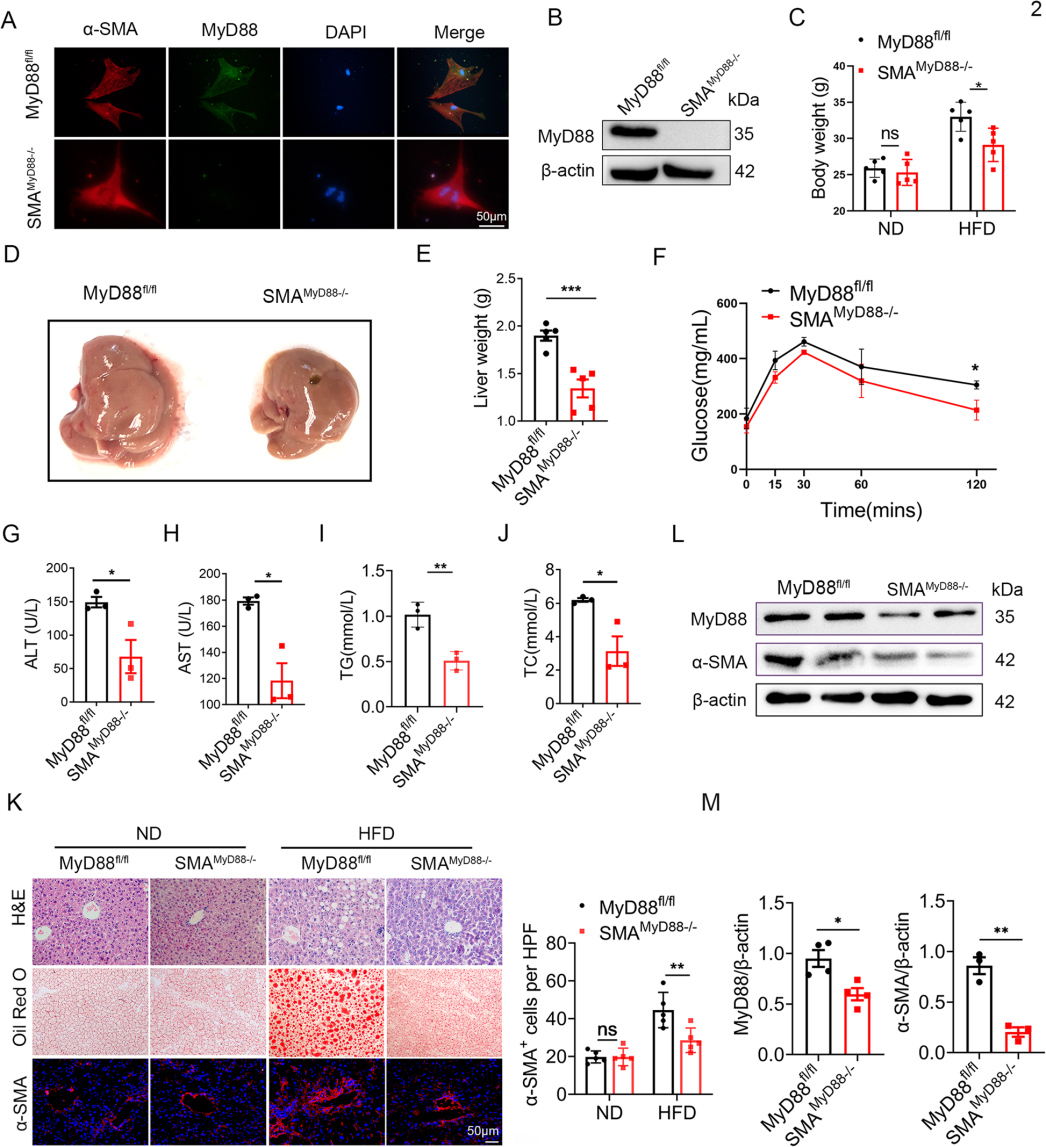
MyD88 deficiency in myofibroblasts attenuated fat accumulation in HFD-induced NAFLD. MyD88 ^fl/fl^ and SMA ^MyD88−/−^ mice were fed with HFD to establish a NAFLD model (*n*  = 5 per group), and the control group was fed with normal diet. The data are representative of at least three independent experiments. **A** Primary hepatic stellate cells were isolated from MyD88 ^fl/fl^ and SMA ^MyD88−/−^ mice liver, immunofluorescence double staining of α-SMA (red) and MyD88 (green) in liver tissues (Scale bar, 50 μm). **B** The protein level of MyD88 in the primary hepatic stellate cells of MyD88 ^fl/fl^ and SMA ^MyD88−/−^ mice were measured using western blot. **C** Body weight at end of diet. **D** Representative images of the livers from SMA ^MyD88−/−^ mice and control mice. **E** Liver weight, (**F**) Glucose tolerance test (GTT), (**G**) ALT, (**H**) AST, (**I**) TG and (**J**) TC levels of MyD88 ^fl/fl^ and SMA ^MyD88−/−^ mice are shown. * *p*  < 0.05, ** *p*  < 0.01. *** *p* < 0.001. **K** Representative H&E, Oil red O staining and staining of α-SMA in liver tissues of MyD88 ^fl/fl^ and SMA ^MyD88−/−^ mice. Scale bar, 50 μm. Statistical analysis. ** *p*  < 0.01 (**L**-**M**) The protein levels of MyD88 and α-SMA in liver tissues of MyD88 ^fl/fl^ and SMA ^MyD88−/−^ mice were determined by western blot. The densities of proteins were quantified using densitometry. Proteins were normalized to β-actin. * *p* < 0.05, ** *p* < 0.01. ALT: alanine aminotransferase; AST: aspartate aminotransferase; TG: triglyceride; TC: total cholesterol

To investigate the role of MyD88 in myofibroblasts, we conditionally deleted MyD88 in murine myofibroblasts as described previously [28, 29]. We crossed mice carrying the loxP-flanked MyD88 allele with α-SMA-cre mice to achieve MyD88 ablation specifically in myofibroblasts (SMA^MyD88−/−^) mice. The absence of MyD88 in the HSCs derived from SMA^MyD88−/−^ mice was confirmed by double immunofluorescence staining (Fig. 2A) and by reduced MyD88 protein levels in HSCs derived from SMA^MyD88−/−^ mice livers compared with those in HSCs derived from MyD88^fl/fl^ mice (Fig. 2B).

To explore the role of MyD88 in NAFLD, SMA^MyD88−/−^ mice and control littermates were used to establish an HFD-induced NAFLD model. We found a significant reduction in the body weight gain and liver weight of SMA^MyD88−/−^ mice compared with those of MyD88^fl/fl^ mice fed with an HFD (Fig. 2C-E). SMA^MyD88−/−^ mice also showed impaired glucose tolerance compared with MyD88^fl/fl^ mice (Fig. 2F). Liver fat accumulation is frequently accompanied with elevated ALT, AST, TG and total TC levels. As shown in Fig. 2G-J, SMA^MyD88−/−^ mice exhibited lower serum levels of ALT, AST, TG and TC. H&E and oil red O staining demonstrated that myofibroblast-specific MyD88 deletion impaired fat accumulation (Fig. 2K). Myofibroblast-specific MyD88 deletion also decreased the expansion of myofibroblasts, as detected via α-SMA immunostaining (Fig. 2K). There were no significant differences between SMA^MyD88−/−^ and MyD88^fl/fl^ mice fed with a normal diet (Fig. 2K). Compared with MyD88^fl/fl^ mice, the downregulated protein levels of MyD88 and α-SMA in the liver tissues of SMA^MyD88−/−^ mice were further confirmed by western blot (Fig. 2L-M). In summary, MyD88 knockout in myofibroblasts significantly attenuated liver injury, and fat accumulation in HFD-induced NAFLD.

### MyD88 deficiency in myofibroblasts attenuated liver inflammation and lipid metabolism-associated gene expression in HFD-induced NAFLD

**Fig. 3:**
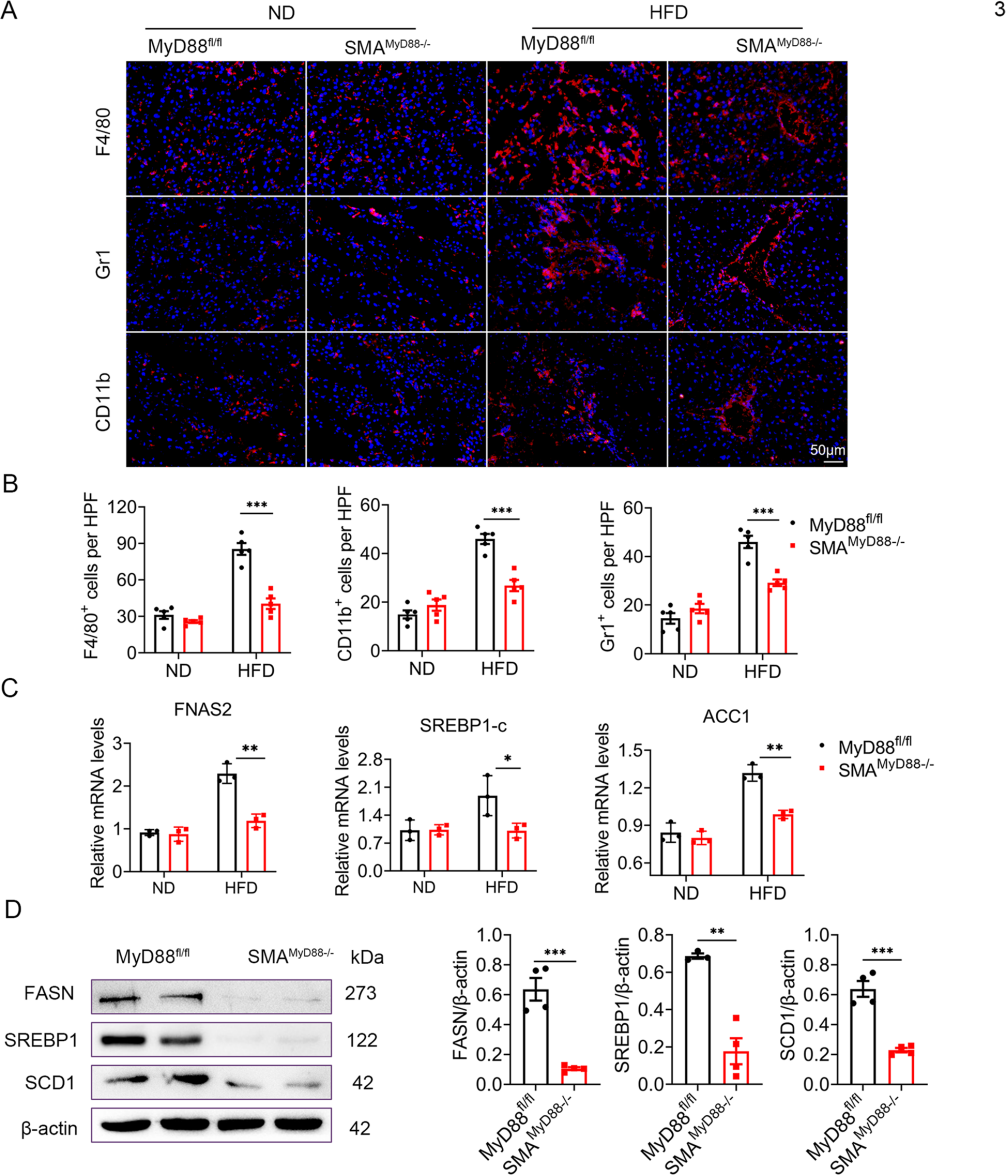
MyD88 deficiency in myofibroblasts attenuated liver inflammation and lipid metabolism-associated gene expression in HFD-induced NAFLD. **A**-**D** MyD88 ^fl/fl^ and SMA ^MyD88−/−^ mice were used for NAFLD model (*n*  = 5 per group), and the control group was fed with normal diet. The data are representative of at least three independent experiments. **A** Representative staining of F4/80, Gr1 and CD11b in liver tissues (Scale bar, 50 μm) and (**B**) Statistical analysis (magnification, 200×). *** *p*  < 0.001. **C** The mRNA levels of FANS2, ACC1 and SREBP1-c in the liver tissues of MyD88 ^fl/fl^ and SMA ^MyD88−/−^ mice were measured using qPCR analysis. ** *p*  < 0.01, *** *p*  < 0.001. **D** The protein levels of FASN, SREBP1 and SCD1 in liver tissue of MyD88 ^fl/fl^ and SMA ^MyD88−/−^ mice were determined by western blot. The densities of proteins were quantified using densitometry. Proteins were normalized to β-actin. ** *p*  < 0.01, *** *p*  < 0.001

SMA^MyD88−/−^ mice showed reduced infiltration of F4/80^+^ macrophages, Gr1^+^ neutrophils and CD11b^+^ monocytes compared with HFD-fed MyD88^fl/fl^ mice. Immune cell infiltration was similar between SMA^MyD88−/−^ and normal diet-fed control mice (Fig. 3A-B). Furthermore, the mRNA levels of FANS2, SREBP1-c and ACC1 were markedly downregulated in the livers of HFD-fed SMA^MyD88−/−^ mice (Fig. 3C). Compared with MyD88^fl/fl^ mice, FASN, SREBP1 and SCD1 protein levels were consistently downregulated in the liver tissues of SMA^MyD88−/−^ mice (Fig. 3D). Therefore, MyD88 knockout in myofibroblasts attenuated liver inflammation and the expression of lipid metabolism-associated genes in HFD-induced NAFLD.

### MyD88 deficiency in myofibroblasts attenuated DEN/HFD-induced NAFLD-related hepatocarcinogenesis

**Fig. 4:**
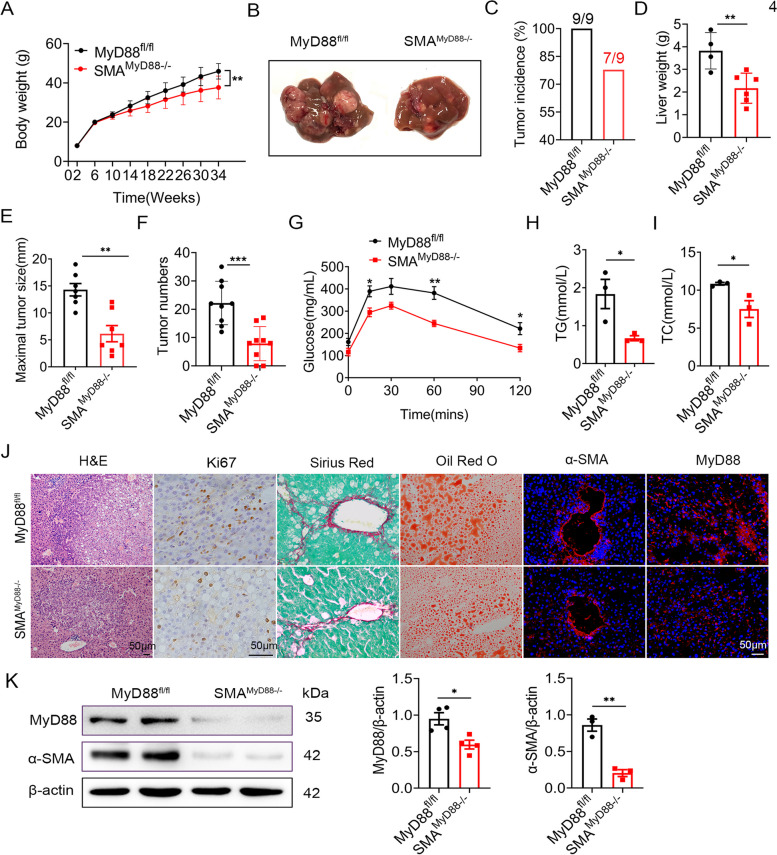
MyD88 deficiency in myofibroblasts attenuated DEN/HFD-induced liver cancer. Groups of MyD88 ^fl/fl^ and SMA ^MyD88−/−^ mice were used for the DEN/HFD-induced liver cancer model (*n*  = 9 per group). The data are representative of at least three independent experiments. **A** Body weight, (**B**) Representative images of mouse livers, (**C**) Tumor incidence, (**D**) Liver weight, **E** Size of the maximal tumors and (**F**) Number of tumors per mouse are shown. * *p*  < 0.05, ** *p*  < 0.01. **G** Glucose tolerance test, serum (**H**) TG and (**I**) TC of MyD88 ^fl/fl^ and SMA ^MyD88−/−^ mice are shown. * *p*  < 0.05, ** *p*  < 0.01. **J** H&E, Ki67, Sirius Red and Oil red O staining and immunofluorescence staining of α-SMA and MyD88 in liver tissues of MyD88 ^fl/fl^ and SMA ^MyD88−/−^ mice. Scale bar, 50 μm. **K** The protein levels of MyD88 and α-SMA in liver tissues of MyD88 ^fl/fl^ and SMA ^MyD88−/−^ mice treated with DEN/HFD were determined by western blot. The densities of proteins were quantified using densitometry. Proteins were normalized to β-actin. *p<0.05, **p<0.01

To explore the role of MyD88 in NAFLD-related liver cancer, SMA^MyD88−/−^ and MyD88^fl/fl^ mice were first treated with an intraperitoneal injection of 50 µg/g DEN (Sigma-Aldrich, St. Louis, MO, USA) at the age of 15 days and 30 days, and then fed with an HFD beginning at 6 weeks of age. Liver tumorigenesis was monitored for 10 months. SMA^MyD88−/−^ mice showed significantly reduced body weight gain (Fig. 4A) All control mice developed liver tumors within 30 weeks; however, SMA^MyD88−/−^ mice showed obvious resistance to liver cancer development; approximately 77.78% of SMA^MyD88−/−^ mice developed tumors (Fig. 4B-C). Myofibroblast-specific MyD88 deletion significantly decreased liver weight, liver tumor size at 10 months and number of liver tumors (Fig. 4D-F). SMA^MyD88−/−^ mice also demonstrated impaired glucose tolerance (Fig. 4G). Compared with control mice, the serum TG and TC levels were significantly decreased in DEN/HFD-treated SMA^MyD88−/−^ mice (Fig. 4H-I). H&E staining, immunohistochemical staining of Ki67, Sirius red staining and Oil red O staining also revealed that DEN/HFD-treated SMA^MyD88−/−^ mice had reduced inflammatory cell infiltration, steatosis, cell proliferation, fibrosis and fat accumulation compared with MyD88^fl/fl^ mice (Fig. 4J). Immunofluorescence staining and western blot indicated that MyD88 and α-SMA expression was significantly downregulated in the liver of SMA^MyD88−/−^ mice compared with that in the liver of DEN/HFD-treated MyD88^fl/fl^ mice (Fig. 4J-K). Thus, MyD88 knockout in myofibroblasts significantly alleviates NAFLD-related hepatocarcinogenesis.

### MyD88 deficiency in myofibroblasts attenuated macrophage M2 polarization and fat accumulation in NAFLD-related HCC

**Fig. 5:**
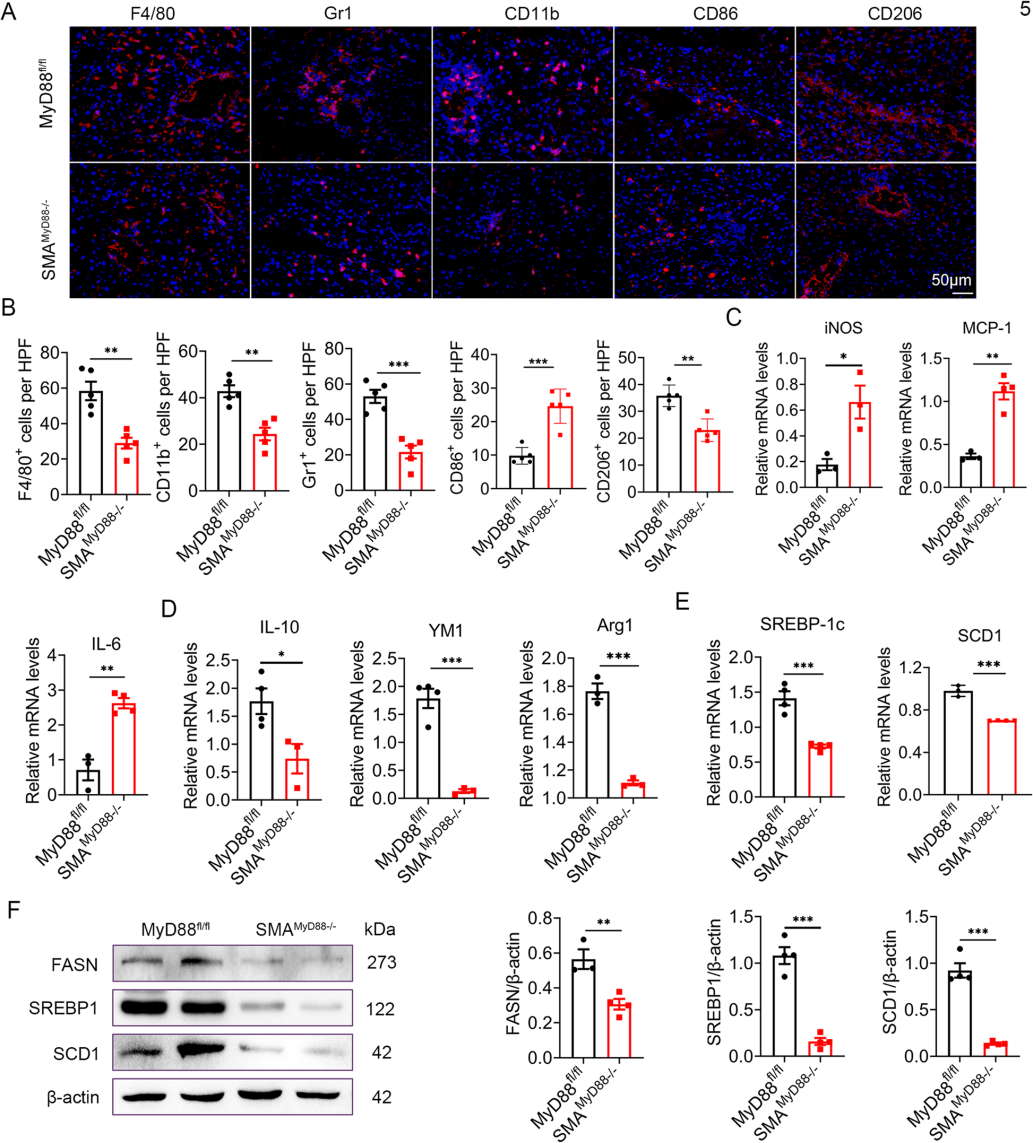
MyD88 deficiency in myofibroblasts attenuated inflammation and fat accumulation in DEN/HFD-induced HCC. Groups of MyD88 ^fl/fl^ and SMA ^MyD88−/−^ mice (*n*  = 9 per group) were used for the DEN/HFD- induced liver cancer model. The data are representative of at least three independent experiments. **A** Representative staining of F4/80, Gr1, CD11b, CD86 and CD206 in liver tissues (Scale bar, 50 μm) and (**B**) Statistical analysis (magnification, 200×). * *p*  < 0.05, ** *p*  < 0.01. **C**-**E** The mRNA levels of iNOS, IL-6, MCP-1, IL-10, Arg1, YM1, SCD1 and SREBP1-c in the liver tissues of MyD88 ^fl/fl^ and SMA ^MyD88−/−^ mice were measured using qPCR analysis. * *p*  < 0.05, ** *p*  < 0.01, *** *p*  < 0.001. **F** The protein levels of FASN, SREBP1 and SCD1 in liver tissues of MyD88 ^fl/fl^ and SMA ^MyD88−/−^ mice were determined by western blot. The densities of proteins were quantified using densitometry. Proteins were normalized to β-actin. *** *p*  < 0.001

Consistent with NAFLD results, MyD88 deletion in myofibroblasts significantly decreased the infiltration of F4/80^+^ macrophages, Gr1^+^ neutrophils, and CD11b^+^ monocytes in NAFLD-related HCC (Fig. 5A-B). HCC is characterized by considerable macrophage infiltration, raising questions about the classification of infiltrating macrophages in liver cancer. We therefore analyzed the number of CD206^+^ (an M2 marker) and CD86^+^ (an M1 marker) macrophages in HCC tissues and revealed that SMA^MyD88−/−^ mice had enhanced M1-like phenotype and reduced M2-like phenotype compared with control mice (Fig. 5A-B).

Furthermore, HCC tissues were analyzed via qPCR for the expression of genes associated with M2-like phenotype, including interleukin-10 (IL-10), arginase-1 (Arg1), and YM1, with those associated M1-like phenotype, including iNOS, interleukin-6 (IL-6), and monocyte chemoattractant protein-1 (MCP-1). Similarly, MyD88 deletion in myofibroblasts upregulated the expression of M1-related genes (Fig. 5C) and downregulated the expression of M2-like factors (Fig. 5D).

We further examined lipogenesis gene expression in HCC tissues. As shown in Fig. 5E, SREBP1-c and SCD1 mRNA levels were markedly downregulated in HCC tissues derived from SMA^MyD88−/−^ mice. Compared with MyD88^fl/fl^ mice, the protein expression levels of FASN, SREBP1, and SCD1 were consistently downregulated in HCC tissues of SMA^MyD88−/−^ mice (Fig. 5F). In summary, MyD88 deletion in myofibroblasts significantly attenuated macrophage M2 polarization and fat accumulation in NAFLD-related HCC.

### Specific deletion of MyD88 in myofibroblasts reduced CCL9 secretion in NAFLD-related HCC

**Fig. 6:**
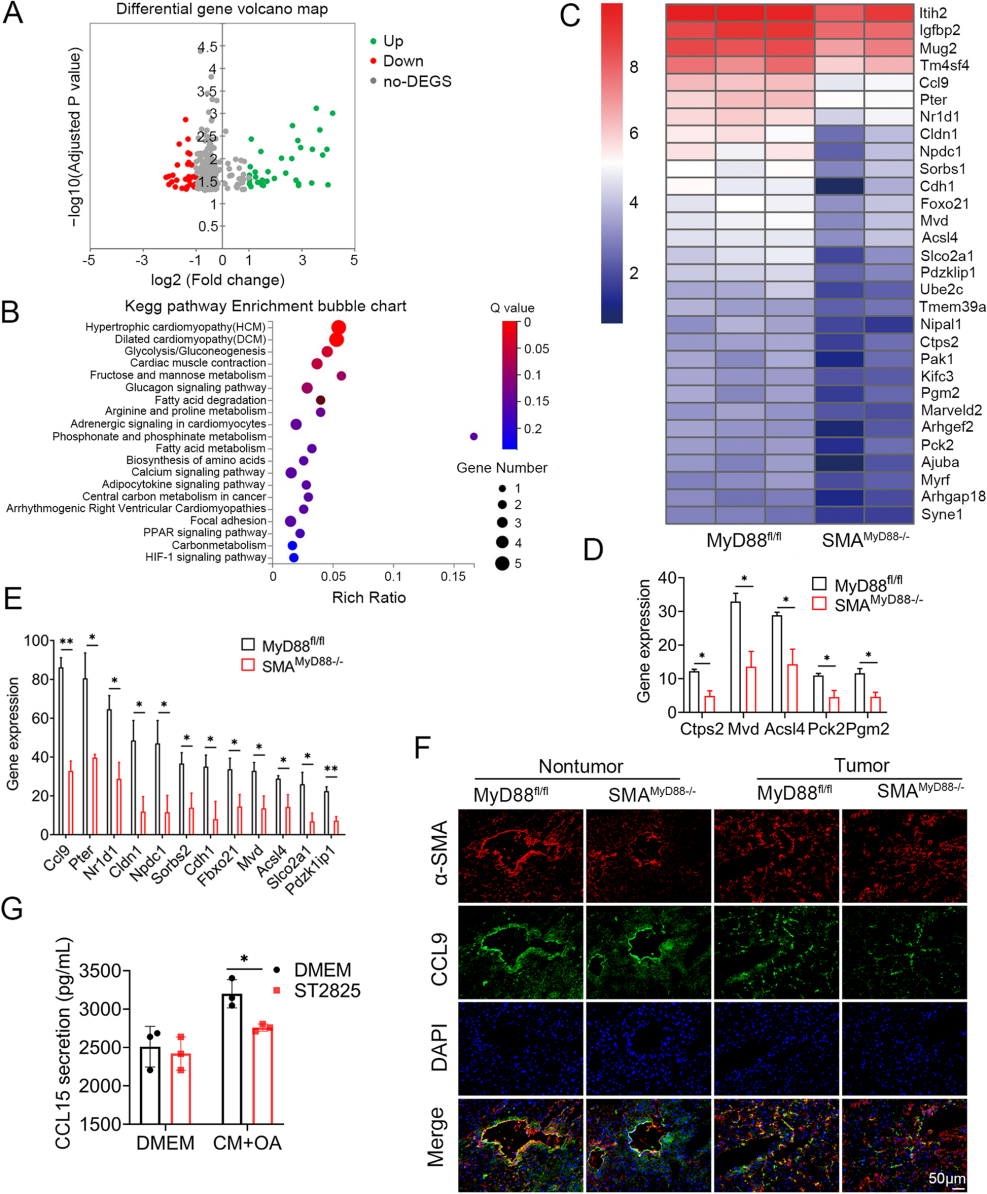
Secreted CCL9 was critical for DEN/HFD induced hepatocarcinogenesis.  RNA sequencing analysis of DEGs between DEN/HFD-induced liver cancer tissues from MyD88 ^fl/fl^ and SMA ^MyD88−/−^ mice. **A** Volcano diagram of DEGs, the threshold is padj < 0.05 and the absolute value of log2 Ratio ≥ 1. **B** KEGG enriched signal pathway analysis with down-regulated genes in liver tissues. **C** Heatmap view of the most significant differential expressed genes. **D** Fat accumulation related gene expression in liver tissues. **E** Gene expression in liver tissues. **F** Immunofluorescence double staining for CCL9 and α-SMA in liver cancer tissues from MyD88 ^fl/fl^ and SMA ^MyD88−/−^ mice. Scale bar. 50 μm. *p<0.05. **G** The protein levels of CCL15 in LX-2 medium were measured using ELISA. *p<0.05

To further identify the gene expression signature, we performed a protein-coding mRNA-seq analysis of HCC tissues derived from DEN/HFD-treated SMA^MyD88−/−^ mice and control mice. A total of 344 differentially expressed genes (DEGs) were identified, including 58 upregulated and 286 downregulated genes (Fig. 6A; padj < 0.05; absolute value of log2 ratio ≥ 1).

A subsequent pathway analysis showed that MyD88 deletion in myofibroblasts mainly promoted the changes in metabolism related pathways, including the metabolism of lipids, amino acids and sugars (Fig. 6B). The expression of genes related to lipid metabolism: Ctps2, Mvd, Acsl4, Pck2, and Pgm2 was significantly downregulated (Fig. 6C-D). Among the 30 genes with the most significant differences in expression, the expression of CCL9, which encodes an inflammatory chemokine, was significantly decreased in DEN/HFD-treated SMA^MyD88−/−^ mice compared with controls (Fig. 6E). Double immunofluorescence staining showed that CCL9 was highly expressed in SMA^+^ cells in the HCC tissues of control mice, indicating that CCL9 was expressed in myofibroblasts. Consistent with the mRNA-seq results, the number of CCL9^+^ cells in the HCC tissues of SMA^MyD88−/−^ mice was significantly decreased (Fig. 6F). CCL9 protein expression levels were further analyzed via ELISA using LX-2-conditioned media (CM). CCL15 (homologous protein CCL9 in humans) secretion, was consistently increased in LX-2 cells induced by HepG2 conditioned media (TCM) and OA; meanwhile, the MyD88 inhibitor ST2825 greatly suppressed TCM and OA-induced CCL15 protein levels in LX-2 cells (Fig. 6G). Thus, myofibroblast-derived CCL9 may play an important role in NAFLD-related HCC.

### CCL9 promoted M2 polarization and fat accumulation in macrophages by activating the STAT6/ PPARβ pathway in HCC

**Fig. 7:**
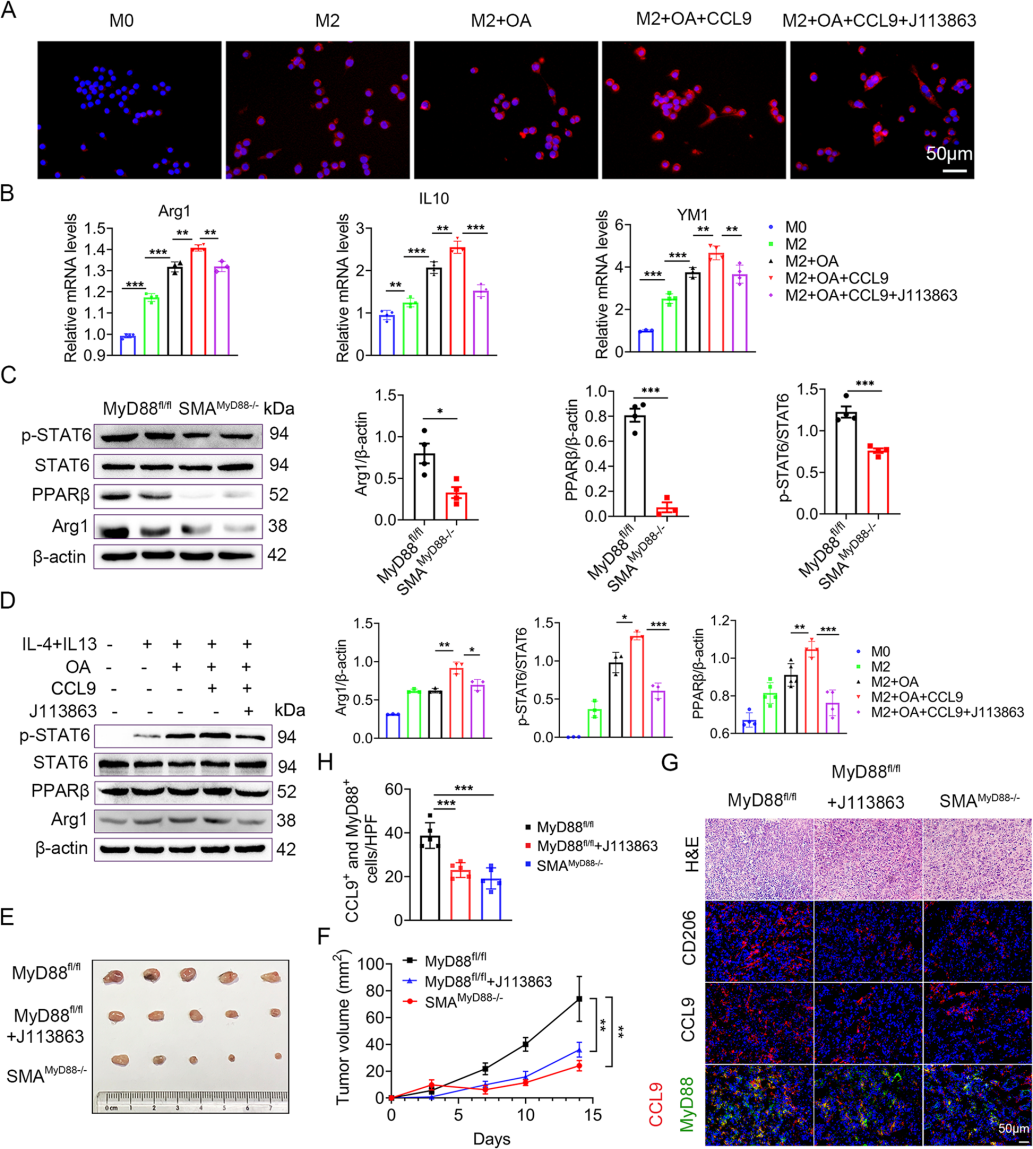
CCL9 promoted M2 polarization in macrophages via activating STAT6/ PPARβ pathway. **A**-**C** RAW264.7 cells was induced by 20 ng/mL IL-4, 20 ng/mL IL-13, 200 µM OA, 20 ng/mL CCL9 recombinant protein, and 500 nM J113863 (inhibitor of CCL9 receptor CCR1) for 48 h. **A** Immunofluorescence staining of CD206 in RAW264.7 cells. **B** mRNA expression of IL-10, Arg1 and YM1 in RAW264.7 cells were detected by real-time PCR method. **C** The protein levels of p-STAT6, STAT6, PPARβ and Arg1 in DEN/HFD induced liver tumors from MyD88 ^fl/fl^ and SMA ^MyD88−/−^ mice were detected by western blot analysis, and the gray value of p-STAT6, STAT6, PPARβ and Arg1 relative expression in livers of mice. PPARβ, Arg1 were normalized to β-actin, p-STAT6 was normalized to STAT6.  **D** The protein levels of p-STAT6, STAT6, PPARβ and Arg1 in RAW264.7 cells were determined by western blot. The densities of proteins were quantified using densitometry. PPARβ, Arg1 were normalized to β-actin, p-STAT6 was normalized to STAT6. * *p*  < 0.05, ** *p*  < 0.01, *** *p*  < 0.001. (E-G) MyD88 ^fl/fl^ and SMA ^MyD88−/−^ mice were fed HFD for 3 months, then 1 × 10 ^6^ Hepa1-6 cells were inoculated subcutaneously in these mice. The mice were fed HFD and blocked by intraperitoneal injection of CCL9 receptor CCR1 inhibitor J113863 (J113863, 3 mg/kg), and tumor growth were evaluated for 2 weeks. **E** Ex vivo images of resected tumors formed by subcutaneous injection of Hepa1-6 cells with or without CCR1 inhibitor (J113863) in MyD88 ^fl/fl^ and SMA ^MyD88−/−^ mice (*n*  = 4 per group). **F** Growth curves of tumor volume. **G** H&E staining, CD206 and CCL9 immunofluorescence staining and and CCL9 and MyD88 immunofluorescence double staining in transplanted tumors. Scale bar, 50 μm. **H** Statistical analysis. *** *p*  < 0.001

We analyzed the HSC-specific role of MyD88 in macrophage polarization in vitro. As MyD88 deletion in myofibroblasts decreased CCL9 secretion, we hypothesized that MyD88 in myofibroblasts promotes the polarization of macrophages through CCL9. RAW264.7 cells were cultured with IL-4 and IL-13 for 48 h to induce M2 polarization. Compared with untreated DMEM group, treatment with IL-4 and IL-13 activated CD206 expression in macrophages. Immunostaining results indicated that OA and CCL9 recombinant proteins further upregulated CD206 expression in macrophages. However, treatment of RAW264.7 cells with J113863 (a CCL9 receptor CCR1 inhibitor) eliminated CCL9-induced expression of CD206 (Fig. 7A). After M2 polarization induction and OA treatment, IL-10, Arg1, and YM1 levels in RAW264.7 cells were significantly upregulated compared to controls. Exogenous addition of CCL9 recombinant protein further promoted M2-related gene expression (Fig. 7B).

Signal transducer and activator of transcription 6 (STAT6) and PPARβ are known to drive macrophage M2 polarization. Higher levels of STAT6 and PPARβ are found in M2 macrophages [30]. To determine how myofibroblast-derived MyD88 affects macrophage polarization, we analyzed HCC tissue and macrophages using western blot. The results revealed that SMA^MyD88−/−^ mice had lower levels of phosphorylated STAT6, PPARβ and Arg1 than MyD88^fl/fl^ mice (Fig. 7C). Consistent with animal experiment results, when IL-4 and IL-13 induced M2 macrophages were cultured with OA, the exogenous CCL9 recombinant protein significantly upregulated p-STAT6 and Arg1 expression (Fig. 7C). In addition, M2 macrophages treated with J113863 simultaneously demonstrated decreased p-STAT6 levels and PPARβ expression (Fig. 7D). These results suggest that CCL9 promotes M2-type polarization of macrophages via the STAT6/PPARβ signaling pathway.

### Blocking of CCL9 inhibited liver tumor growth by preventing M2 polarization of macrophages in the transplanted tumor model

To further explore the role of CCL9 in liver cancer progression, MyD88^fl/fl^ and SMA^MyD88−/−^ mice were used to establish the subcutaneous transplantation model for Hepa1-6 cells. The mice were fed with HFD for 3 months, after which 1 × 10^6^ Hepa1-6 cells were subcutaneously in these mice. The mice were fed with HFD and blocked by intraperitoneal injection of CCL9 receptor CCR1 inhibitor J113863, Tumor growth was evaluated for 2 weeks. MyD88 knockout in myofibroblasts significantly inhibited tumor growth. Similar effects were observed in the CCR1 inhibition group (Fig. 7E-F). When M2 polarization was analyzed using CD206 immunofluorescence staining, we found that SMA^MyD88−/−^ and J113863-injected MyD88^fl/fl^ mice had attenuated M2-type polarization compared with MyD88^fl/fl^ mice. Furthermore, SMA^MyD88−/−^ mice, showed decreased CCL9 expression compared with MyD88^fl/fl^ mice as determined via CCL9 immunofluorescence staining (Fig. 7G). In addition, the numbers of CCL9^+^ /MyD88^+^ cells were significantly decreased in SMA^MyD88−/−^ and J113863-injected MyD88^fl/fl^ mice (Fig. 7G-H). We revealed that, MyD88 in myofibroblasts promotes M2 polarization via CCL9 secretion in transplanted tumors. Simultaneously, inhibited the CCL9 receptor CCR1 to block CCL9, exerting an antitumor effect.

### CCLl5 expression was increased in myofibroblasts of HCC and was associated with shorter survival of patients with HCC

**Fig. 8:**
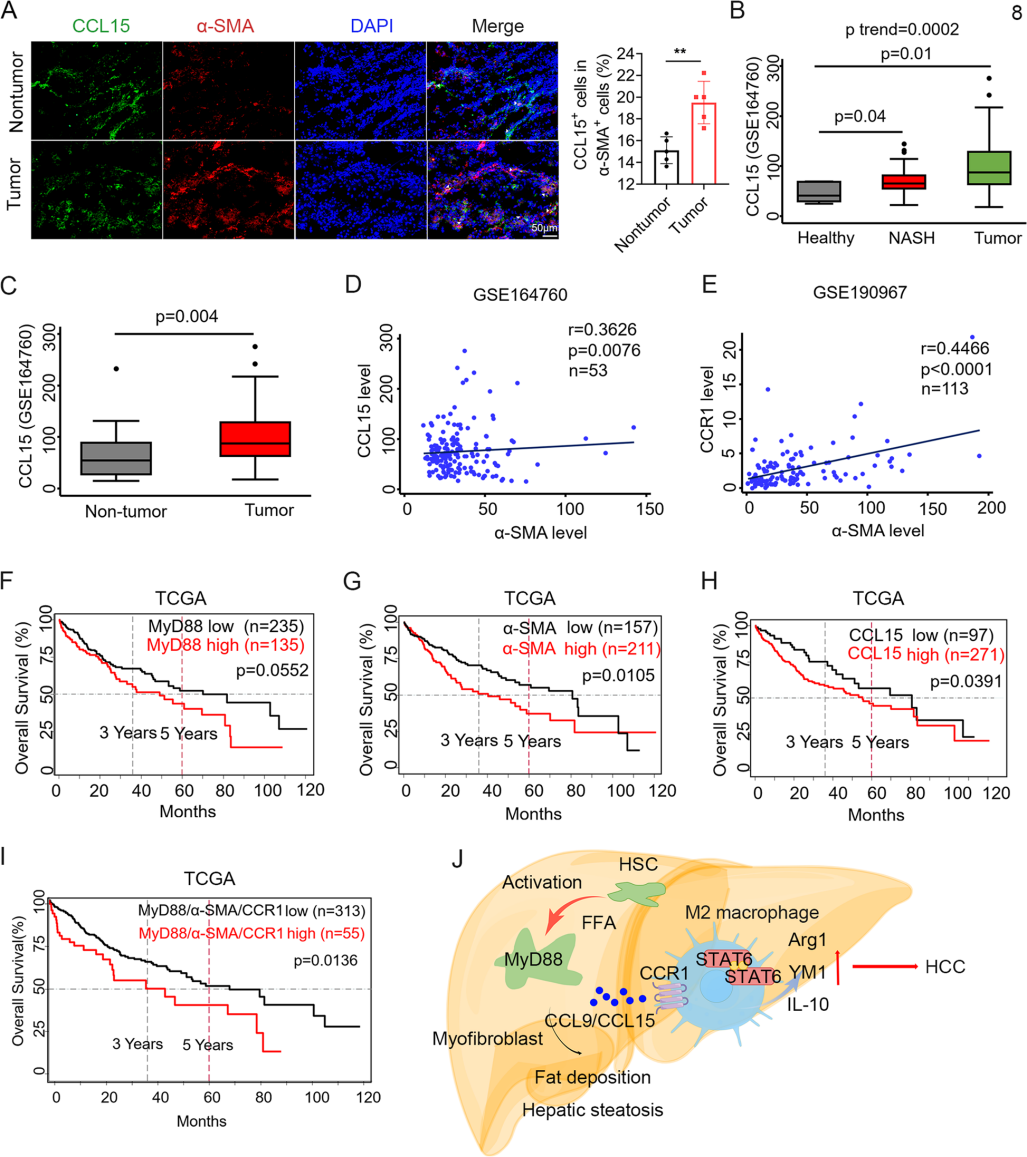
Myofibroblast dependent CCL15 is associated with shorter survival of patients with HCC. **A** Double staining of MyD88 and CCL15 in tumor tissues and adjacent paracancerous tissues from HCC samples. Scale bar, 50 μm. **B**-**C** Boxplots showing the expression levels of CCL15 in the liver dataset GSE164760. **B** Healthy: *n*  = 6; NASH: *n*  = 74; Tumor: *n*  = 53. **C** Nontumor: *n*  = 29; Tumor: *n*  = 53. **D**-**E** Spearman’s correlation analysis showed correlations among α-SMA, CCL15 and CCR1 in the liver dataset GSE164760 and GSE190967. **F**-**I** Kaplan-Meier curve for survival in the HCC TCGA dataset with MyD88, α-SMA and CCL15, combined MyD88/α-SMA/CCR1 gene expression bifurcated by the spline method to group into low vs. high. Three years and five years are indicated as dotted lines. **J** Schematic of MyD88 signaling in myofibroblasts in the model of NAFLD-related HCC

To extend our findings to human samples, we performed immunofluorescence double staining of α-SMA and CCL15 in tumor tissues and adjacent paracancerous tissues derived from patients with HCC. Compared with paracancerous tissues, the number of α-SMA^+^ fibroblasts expressing CCL15 evidently increased in tumor tissues (Fig. 8A). Furthermore, we analyzed the publicly available GEO dataset GSE164760, and compared the CCL15 expression of healthy, NASH and HCC tissues. Compared with healthy controls, CCL15 mRNA levels were significantly increased in NASH and HCC tissues. CCL15 expression level in tumor tissues was also higher than that in HCC paracancerous tissues. Next, we found significant positive correlations among CCL15, CCR1 and α-SMA in HCC by correlation analysis with GEO data set GSE164760 and GSE190967 (Fig. 8C-E). To further support the α-SMA-MyD88-CCL15 axis in HCC, we performed survival analysis using RNA-seq data acquired from the TCGA database (http://cancergenome.nih.gov/). We found that the high level of α-SMA, MyD88 or CCL15 significantly impaired the overall survival compared with their corresponding low levels (Fig. 8F-H). In addition, a combined high expression level of α-SMA-MyD88-CCR1 significantly impaired overall survival compared with their low expression level (Fig. 8I). Altogether, these results suggest that the upregulation of MyD88 and CCL15 in myofibroblasts is a poor prognostic factor of HCC.

## Discussion

In this study, we explored the role of the MyD88 signaling pathway in HSCs/myofibroblasts during the development from NAFLD to HCC. MyD88 deficiency in myofibroblasts attenuated fat accumulation in HFD-induced NAFLD and NAFLD-related HCC. This study showed that MyD88 in myofibroblasts promotes CCL9/CCL15 secretion, which enhanced macrophage M2 polarization by activating the STAT6/PPARβ pathway to promote hepatocarcinogenesis (Fig. 8J).

Previous studies have shown that MyD88 plays a key role in in obesity-associated NAFLD. However, the relevant molecular mechanisms remain unknown. Complete deletion of MyD88 in HFD-fed mice resulted in obesity, IR, and hepatic steatosis [18, 19]. Hepatocyte MyD88 affected bile acids, gut microbiota, and the metabolome, thereby regulating glucose and lipid metabolism [20]. In contrast, MyD88 depletion in myeloid cells and intestinal epithelial cells reduced diet-induced obesity, systemic inflammation, and insulin resistance [21, 22]. In this study, we used HSCs/myofibroblasts-specific MyD88-deficient mice and found that specific deletion of MyD88 in HSCs/myofibroblasts attenuated hepatic steatosis, inflammatory cell infiltration, and adipogenesis in an HFD-induced NAFLD mouse model (Fig. 2). These novel findings demonstrate the MyD88-dependent function of HSCs in NAFLD. Using alcoholic fatty liver disease model, we consistently revealed that MyD88 in HSCs promotes hepatic adipogenesis and inflammatory responses via OPN secretion [31].

Toll-like receptor signaling through MyD88 contributed to hepatocarcinogenesis. MyD88 deficient mice developed fewer and smaller HCC tumors than WT mice in DEN-induced hepatocarcinogenesis [23]. In hepatocytes, MyD88 signaling promoted HBV-mediated liver carcinogenesis [32]. In Kupffer cells, resulted in secretion of IL-6 which contributed development of DEN-induced hepatocarcinogenesis [23]. Recently, we also found that MyD88 in HSCs/myofibroblasts promoted the progress of DEN/CCl_4_-induced fibrosis-related HCC via CCL20 secretion and promoted the aerobic glycolysis of liver cancer cells [28]. Although MyD88 is widely expressed by different cells, the cell type-specific role of MyD88 in NAFLD-related HCC is mostly unknown. Antje Mohs et al. investigated the role of MyD88 during progression from NASH to HCC using a mouse model of chronic liver injury model. They found that MyD88 deficiency impaired HCC formation; however, hepatocyte specific MyD88 deletion did not affect disease progression. These results suggested that MyD88 in non-parenchymal liver cells was necessary for carcinogenesis during chronic liver injury [33]. The current study revealed that the specific deletion of MyD88 in HSCs/myofibroblasts attenuated DEN/HFD-induced hepatocarcinogenesis (Fig. 4), hepatic steatosis, cell proliferation, and lipogenesis (Fig. 5). These findings were consistent with those of the NAFLD model. This is the first report to describe the myofibroblast-specific role of MyD88 signaling in NAFLD-related HCC. Myofibroblast-specific MyD88 promotes NAFLD-related HCC.

CAFs are the most important stromal components in HCC and are crucial for HCC development and progression, since more than 80% of HCCs develop in fibrotic or cirrhotic livers [11, 34]. CAFs in HCC are mostly derived from HSCs, and α-SMA is a commonly used marker for CAFs [35]. Activated HSCs promote HCC progression by secreting cytokines such as HGF and CCL20 [28, 36] and crosstalk with hepatocytes, macrophages, and other stromal cells [28, 34, 37]. The progression of NAFLD to NASH and liver fibrosis is closely associated with a series of liver injury resulting from lipotoxicity, oxidative stress, or ER stress. These injuries activate HSCs, enhance collagen deposition, and promote liver fibrosis and cirrhosis [38, 39]. In addition, altered ECM composition in fibrotic or cirrhotic NASH livers contributes to carcinogenesis [40, 41]. However, the detailed mechanism of HSC in NAFLD-related HCC has not been elucidated. We found that MyD88 in HSCs promotes macrophage M2 polarization and enhances the progression of NAFLD to HCC by secreting CCL9 (Fig. 8J).

TAMs are the most abundant immune cells in the TME and exert import anti-tumor or pro-tumor effects depending on the balance of macrophage M1/M2 polarization [42, 43]. In HCC, TAMs promote tumor growth, angiogenesis, invasion, and metastasis by releasing cytokines, chemokines, and matrix metalloproteinases (MMPs). In HCC, crosstalk between activated HSCs and macrophages showed that interaction with CD68 and regulation of GAS6 expression by endosialin in fibroblasts drives recruitment and polarization of macrophages in HCC [37].

CCL9, a chemokine also known as macrophage inflammatory protein-1 gamma (MIP-1γ), is often produced in macrophages, osteoclasts, and mesenchymal stem cells [44, 45]. CCL9/CCR1 signaling is important for macrophage recruitment, angiogenesis, and tumor cell invasion [46–48]. In this study, MyD88 in HSCs enhanced CCL9 secretion and further promoted macrophage M2 type polarization via activating the STAT6/ PPARβ pathway (Fig. 7). Blocking CCL9 inhibited Hepa1-6 tumor growth, further demonstrating that CCL9 is a key downstream molecule of MyD88 in myofibroblasts in obesity-related liver cancer (Fig. 7). However, owing to the wide range of cytokines secreted by myofibroblasts, it remains unclear whether other cytokines derived from myofibroblasts contribute to NAFLD-related HCC development.

In conclusion, MyD88 plays a promoting role in myofibroblasts in NAFLD and NAFLD-related HCC. MyD88 in myofibroblasts promotes fat accumulation by enhancing lipid droplet accumulation in HSCs and promotes macrophage M2 polarization and enhances NAFLD to HCC progression by secreting CCL9/CCL15. MyD88 in HSCs/myofibroblasts may be a potential therapeutic and/or preventative target for NAFLD and HCC.

### Supplementary Information

**Additional file 1.**

**Additional file 2.**

## Abbreviations

NAFLD: Nonalcoholic fatty liver disease
HCC: Hepatocellular carcinoma
NASH: Nonalcoholic steatohepatitis
MyD88: Myeloid differentiation factor 88
TLRs: Toll-like receptors
DEN: Diethylnitrosamine
HFD: High-fat diet
ALT: Serum alanine aminotransferase
AST: Aspartate aminotransferase
TG: Triglyceride
TC: Total cholesterol
