# Supplementary material for: MyD88 in myofibroblasts enhances nonalcoholic fatty liver disease-related hepatocarcinogenesis via promoting macrophage M2 polarization
Source: Cell Commun Signal. 2024 Jan 30;22:86. doi: 10.1186/s12964-024-01489-x (PMC10826060; doi:10.1186/s12964-024-01489-x)
Supplement: Supplementary file 1 — Additional file 1. [file 12964_2024_1489_MOESM1_ESM.docx]

**Materials and Methods**

**Blood biochemical assays**

Blood samples of mice were centrifuged at 3000 rpm for 8 min to obtain serum. The levels of serum alanine aminotransferase (ALT), aspartate aminotransferase (AST), triglyceride (TG) and total cholesterol (TC) were detected by Beijing Vital River Laboratory Animal Technology (Beijing, China).

For the glucose tolerance test (GTT), blood samples were obtained at 0, 15, 30, 60 and 120 min after intraperitoneal injection of 2 g/kg dextrose. Blood glucose values were determined using Accu-Chek Performa glucometer (Roche, Basel, Switzerland).

**Histology and immunostaining**

Paraffin or cryostat sections of liver tissue were prepared as described previously[22]. The sliced liver paraffin sections were stained with hematoxylin and eosin (H&E) (Zhongshanjinqiao, Beijing, China). To detect hepatic fat accumulation, cryostat liver sections were stained with Oil Red O (Baso, Zhuhai, Guangzhou, CN). For immunohistochemistry (IHC), cryostat sections were incubated with anti-Ki67 antibodies (BD Pharmingen, San Diego, CA, USA) followed by incubation with horseradish peroxidase (HRP)-conjugated secondary antibodies. For fluorescence staining, cryostat sections were incubated with anti-F4/80, anti-CD11b, anti-Gr1, anti-CD86 (Santa Cruz Biotechnology, TX, USA), anti-CD206 antibodies (BD Pharmingen, San Diego, CA, USA), anti-MyD88 and anti-α-SMA antibodies (Abcam, Cambridge, Cambs, UK) followed by incubation with Alexa Fluor 488-conjugated or Alexa Fluor 594-conjugated secondary antibodies (1:500; Invitrogen, Carlsbad, CA, USA). Sections were evaluated under a micro-scope (DP71, OLYMPUS, Tokyo, Japan) for bright-field and fluorescence microscopy.

**Mouse transplanted tumor model**

Groups of MyD88^fl/fl^ and SMA^MyD88-/-^ mice were fed with HFD for 3 months. Exponentially growing Hepa1-6 cells were harvested and washed, and a suspension of 1×10^6^ cells in 200 μl of sterilized phosphate-buffered saline was injected subcutaneously into the abdomen region of MyD88^fl/fl^ and SMA^MyD88-/-^ mice. The mice were continued fed with HFD. Two days later, the mice received vehicle or 3 mg/kg CCR1 inhibitor J113863 by intraperitoneal injection twice a week for 2 weeks. Tumor growths were measured for 2 weeks.

**Cell lines and treatments**

The LX-2 cell line was purchased from Xiangya Medical College (Changsha, China), and RAW264.7 cell line was obtained from the American Type Culture Collection (ATCC; TIB-71, VA, USA). These cells were cultured in Dulbecco’s modified Eagle’s medium (DMEM)/1640 containing 10% fetal bovine serum (FBS) and 1% penicillin/streptomycin at 37°C with 5% CO_2_. The LX-2 cells were exposed to ST2825 (10 μM, MedChemExpress, Princeton, NJ, USA) for 2 h. After incubation, the cells were challenged with OA (200 μM, Sigma-Aldrich, St. Louis, MO, USA) for 24 h for further analysis. RAW264.7 cells were induced by 20 ng/mL IL-4, 20 ng/mL IL-13, 200 μM OA, 20 ng/mL CCL9 recombinant protein, and 500 nM J113863 (inhibitor of CCL9 receptor CCR1) for 48 h.

**RNA Sequencing Analysis**

RNA-sequencing analyses were performed in DEN/HFD-induced HCC tissues from control and SMA^MyD88-/-^ mice. Total RNA was extracted with RNeasy Mini Kit (QIAGEN, Dusseldorf, Germany), and RNA-sequencing analyses were performed on the BGISEQ-500 sequencer platform by BGI (Shenzhen, China). Stats package and plots with ggplot2 (RRID:SCR_014601) package in R (version 3.5) were used in principle component analysis. The raw transcriptomic reads were mapped to Nipponbare reference genome using HISAT40/Bowtie241 tools after removing adaptor sequences, reads containing polyN sequences, and low-quality reads. Normalization was performed and RESM software was used. Significantly differentially expressed genes (DEGs) were identified by setting padj <0.05, and the absolute value of log2 Ratio ≤ 0.5. The KEGG (Kyoto Encyclopedia of Genes and Genomes) enrichment analysis was performed by using phyper in R. All data mining, and figure presentation were conducted on the Dr Tom network platform of BGI (<http://report.bgi.com>).

Table S1. Real-time PCR primer sequences.

| Gene | [primer sequence](javascript:;) |
| --- | --- |
| Cre F | GATCTCCGGTATTGAAACTCCAGC |
| Cre R | GCTAAACATGCTTCATCGTCGG |
| MyD88 F | GCTCCTCTTAGGGGCCACT |
| MyD88 R | CCACGTCTCACCATTGGGG |
| α-SMA F | GTCCCAGACATCAGGGAGTAA |
| α-SMA R | TCGGATACTTCAGCGTCAGGA |
| FANS2 F | ACCTCCAGTCGTGAAACCAT |
| FANS2 R | CTCAGCTGTGTCTTGGATGC |
| SREBP1 F | GGAGTGGGTAAACTGAGGCT |
| SREBP1 R | TTTGATCCCGGAAGCTCTGT |
| SCD1 F | GCAAGCTCTACACCTGCCTCTT |
| SCD1 R | CGTGCCTTGTAAGTTCTGTGGC |
| iNOS F | CGGAGATCAATGTGGCTGTG |
| iNOS R | GAAGGACTCTGAGGCTGTGT |
| IL-6 F | TTCTTGGGACTGATGCTGGT |
| IL-6 R | CTGTGAAGTCTCCTCTCCGG |
| MCP-1 F | GCTACAAGAGGATCACCAGCAG |
| MCP-1 R | GTCTGGACCCATTCCTTCTTGG |
| TNF-α F | TGAGGTCAATCTGCCCAAGT |
| TNF-α R | GGGGTCAGAGTAAAGGGGTC |
| IL-12p40 F | GACATGTGGAATGGCGTCTC |
| IL-12p40 R | TTATTCTGCCGTGCTTC |
| Arg1 F | CTCCAAGCCAAAGTCCTTAGAG |
| Arg1 R | AGGAGCTGTCATTAGGGACATC |
| IL-10 F | GCTCTTACTGACTGGCATGAG |
| IL-10 R | CGCAGCTCTAGGAGCATGTG |
| YM1 F | CTCAACCTGGACTGGCAGTA |
| YM1 R | CTGCTCCTGTGGAAGTGAGT |
| β-actin F | ACCAGTTCGCCATGGATGAC |
| β-actin R | TGCCGGAGCCGTTGTC |

F: forward primer; R: reverse primer
