# Supplementary material for: MyD88 in myofibroblasts enhances nonalcoholic fatty liver disease-related hepatocarcinogenesis via promoting macrophage M2 polarization
Source: Cell Commun Signal. 2024 Jan 30;22:86. doi: 10.1186/s12964-024-01489-x (PMC10826060; doi:10.1186/s12964-024-01489-x)
Supplement: Supplementary file 2 — Additional file 2. [file 12964_2024_1489_MOESM2_ESM.docx]

Original Western Blot

Fig.1E


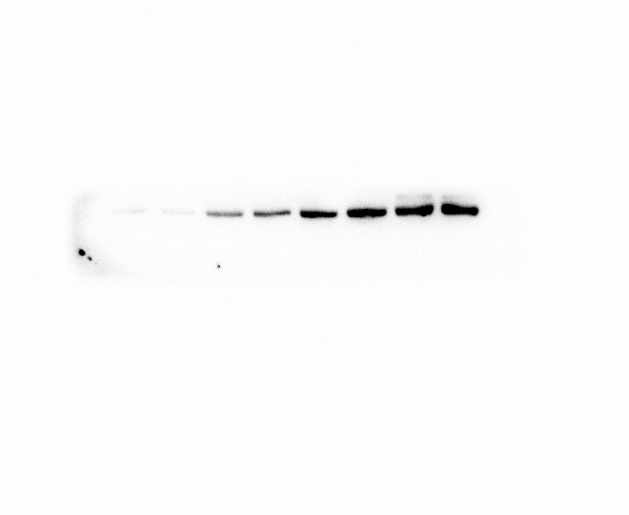


α-SMA

42KD


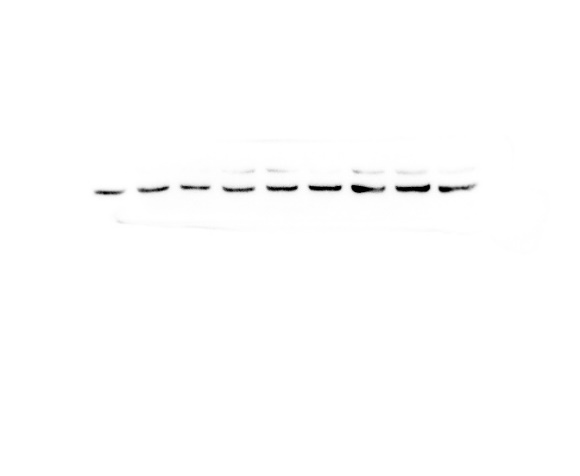


MyD88

42KD


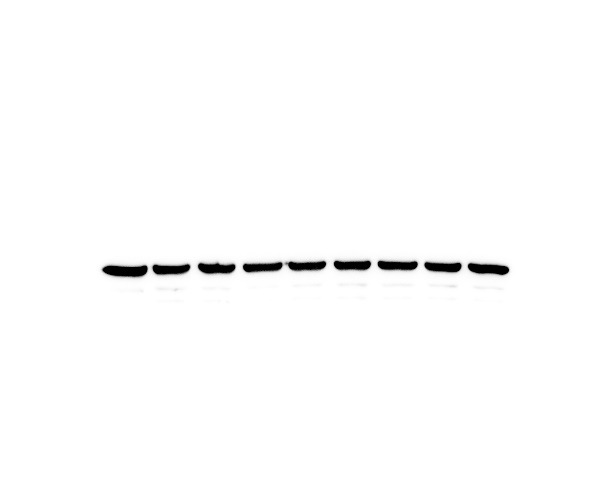


β-actin

42KD

Fig2B


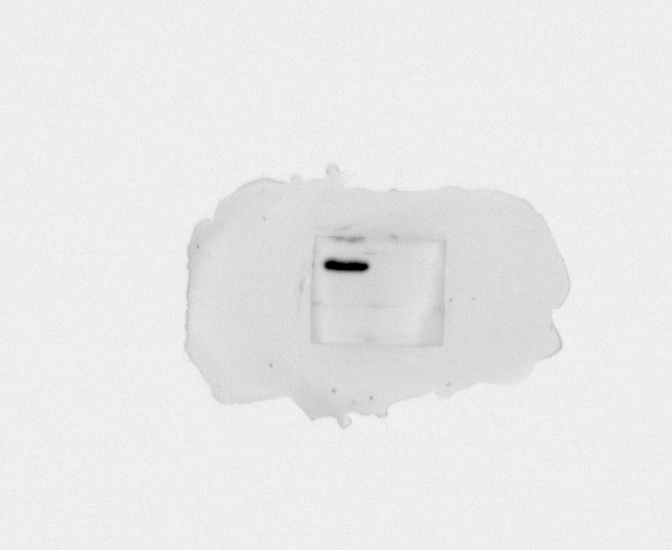


MyD88

42KD


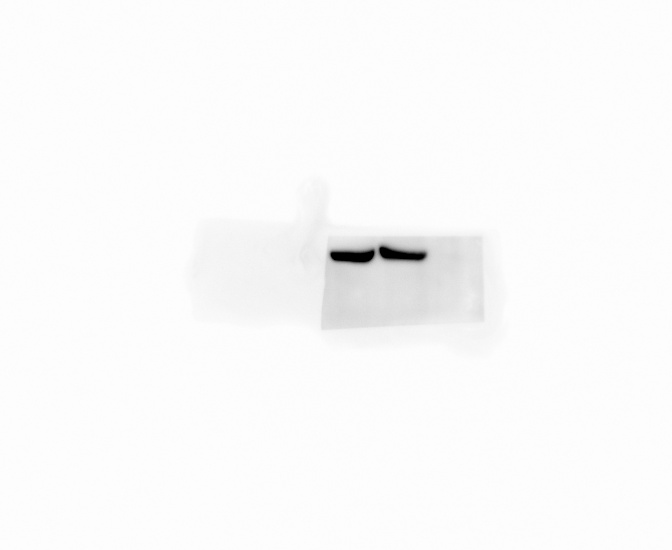


β-actin

42KD

Fig.2L


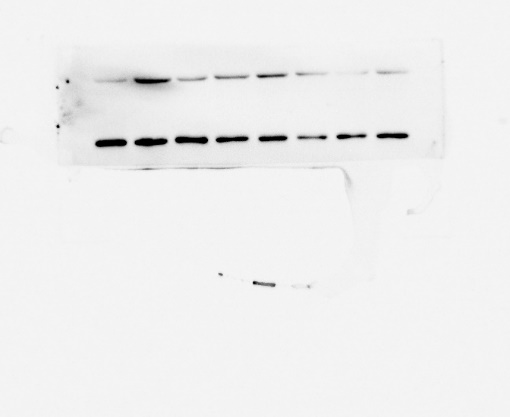


MyD88

42KD


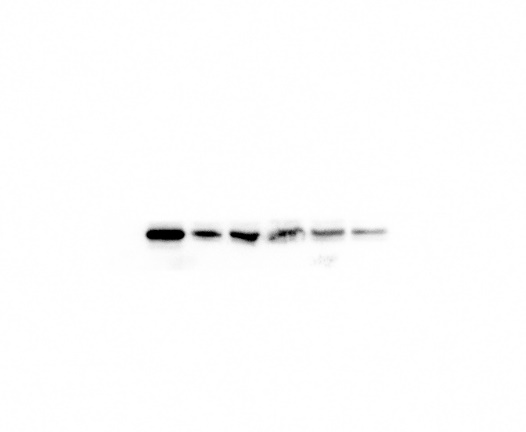


α-SMA

42KD


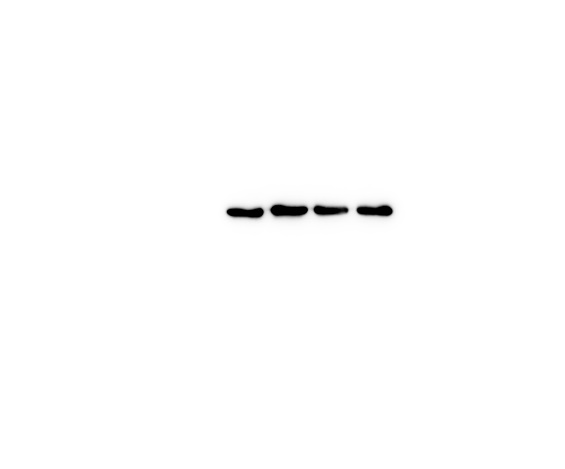


β-actin

42KD

Fig.3D


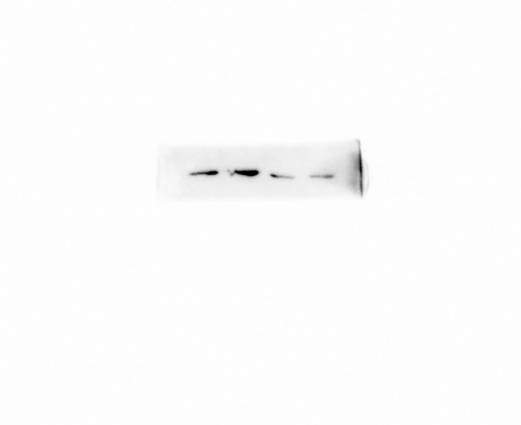


SCD1

42KD


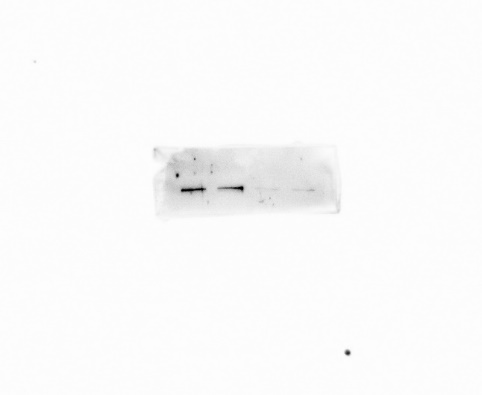


FASN

273KD


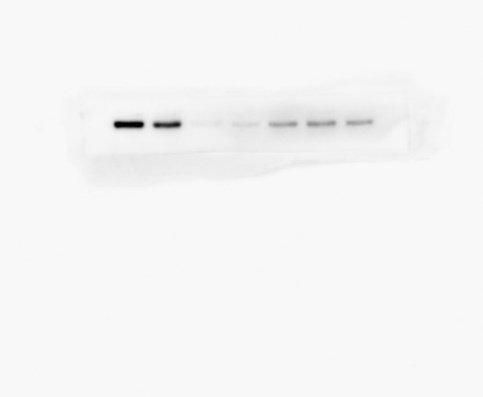


SREBP1

122KD


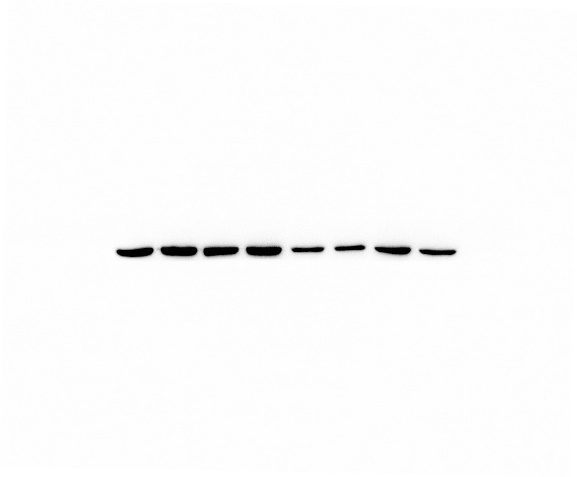


β-actin

42KD

Fig.4J


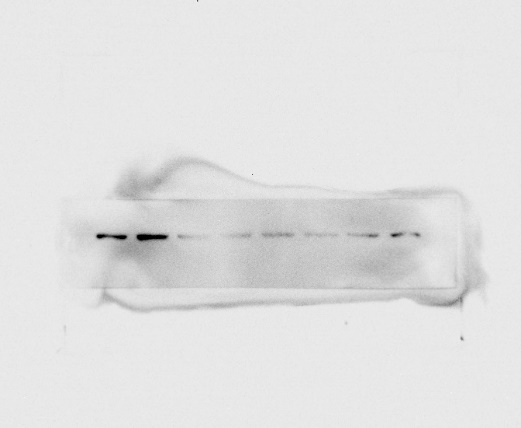


MyD88

42KD


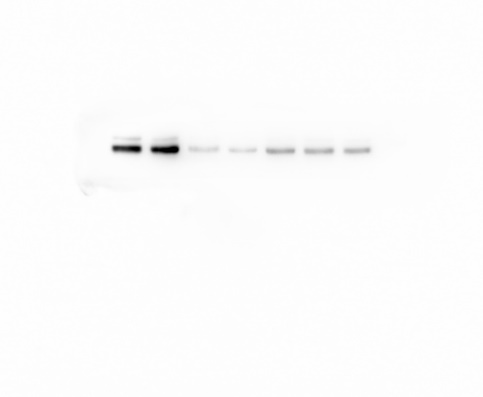


α-SMA

42KD


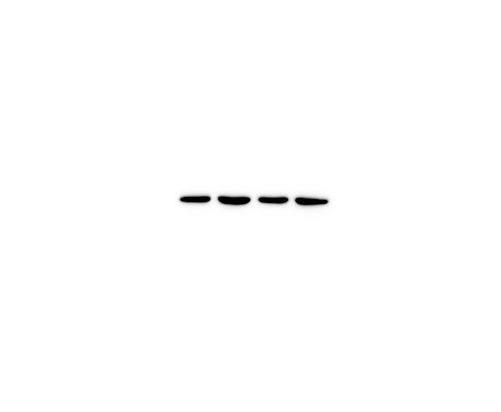


β-actin

42KD

Fig.5F


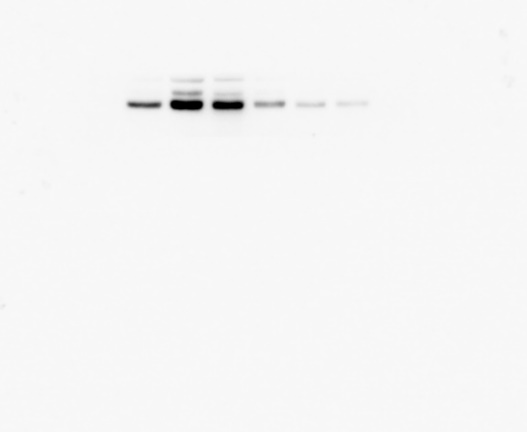


SREBP1

122KD


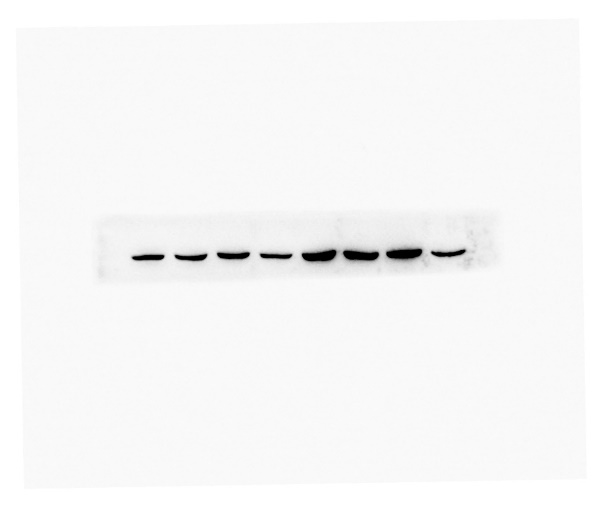


β-actin

42KD


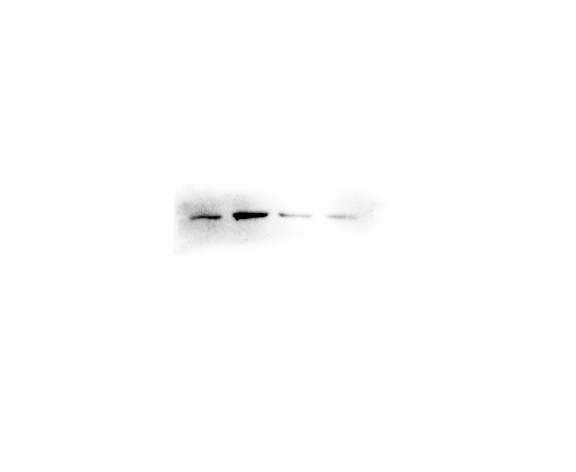


SCD1

42KD


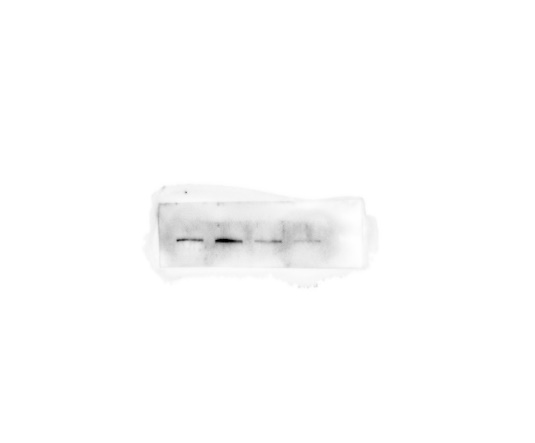


FASN

273KD

Fig.7C


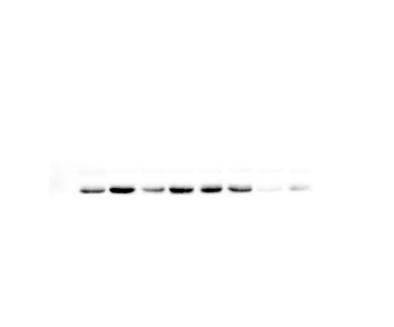


PPARβ

52KD


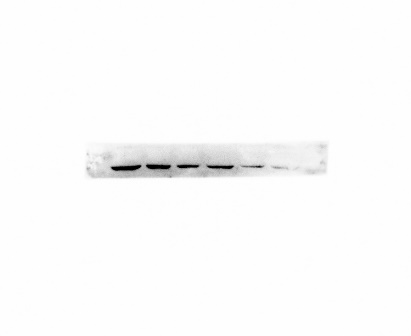


p-STAT6

94KD


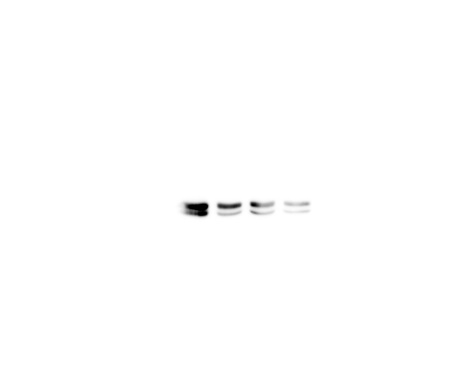


Arg1

94KD


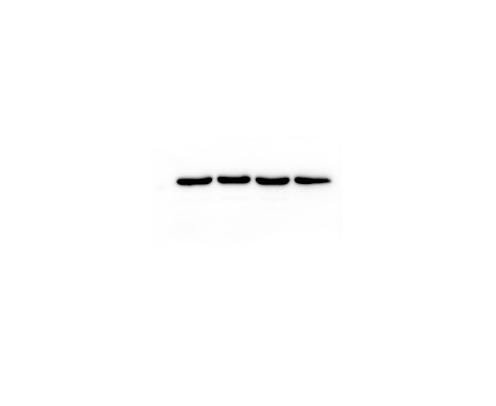


β-actin

42KD


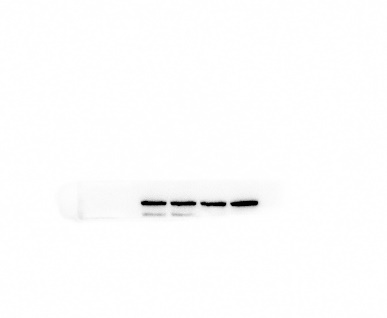


STAT6

94KD

Fig.7D


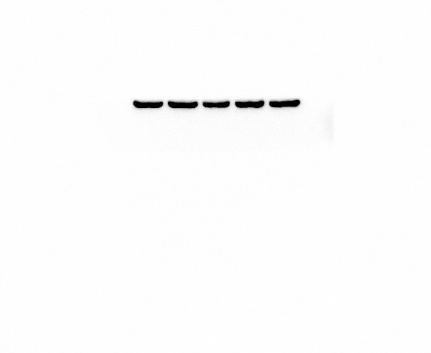


β-actin

42KD


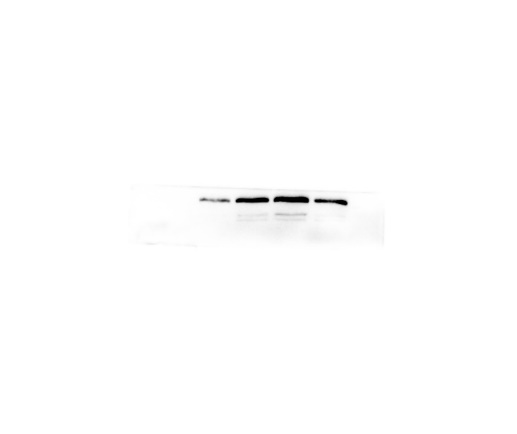


p-STAT6

94KD


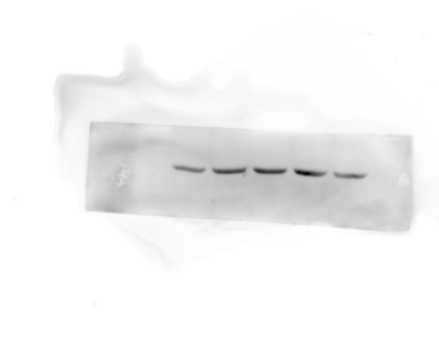


Arg1

94KD


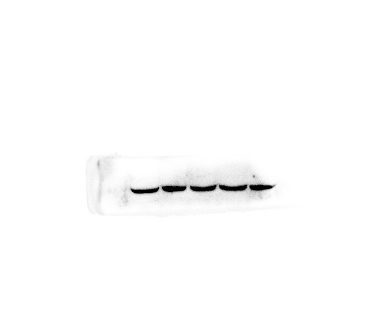


PPARβ

52KD


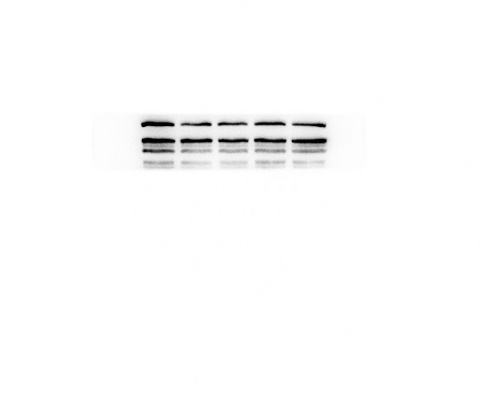


STAT6

94KD
